# CircPLEKHM3 acts as a tumor suppressor through regulation of the miR-9/BRCA1/DNAJB6/KLF4/AKT1 axis in ovarian cancer

**DOI:** 10.1186/s12943-019-1080-5

**Published:** 2019-10-17

**Authors:** Lei Zhang, Qing Zhou, Qiongzi Qiu, Ling Hou, Mengting Wu, Jia Li, Xufan Li, Bingjian Lu, Xiaodong Cheng, Pengyuan Liu, Weiguo Lu, Yan Lu

**Affiliations:** 1 Center for Uterine Cancer Diagnosis & Therapy Research of Zhejiang Province, Women’s Reproductive Health Key Laboratory of Zhejiang Province, and Department of Gynecologic Oncology, Women’s Hospital, Zhejiang University School of Medicine, Hangzhou, 310029 Zhejiang China; 2 Institute of Translational Medicine, Zhejiang University School of Medicine, Hangzhou, China; 3 Department of Respiratory Medicine, Sir Run Run Shaw Hospital, Zhejiang University School of Medicine, Hangzhou, China; 4 Department of Physiology and Center of Systems Molecular Medicine, Medical College of Wisconsin, Milwaukee, WI USA

**Keywords:** CircPLEKHM3, Ovarian cancer, miR-9, BRCA1, DNAJB6, KLF4, AKT1, Prognosis

## Abstract

**Background:**

Emerging evidence has shown that circular RNAs (circRNAs) play essential roles in cancer biology and are potential biomarkers and targets for cancer therapy. However, the expression and function of circRNAs in ovarian carcinogenesis and its progression remain elusive.

**Methods:**

RNA sequencing was performed to reveal circRNA expression profiles in ovarian cancerous and normal tissues. Single-molecule RNA in-situ hybridization was used to quantify circPLEKHM3 expression in tumor tissues. Cell-based in-vitro and in-vivo assays were subsequently conducted to support the clinical findings.

**Results:**

CircPLEKHM3 was identified as one of the most significantly down-regulated circRNAs in ovarian cancer tissues compared with normal tissues. Its expression was further decreased in peritoneal metastatic ovarian carcinomas compared to primary ovarian carcinomas. Patients with lower circPLEKHM3 tend to have a worse prognosis. Functionally, circPLEKHM3 overexpression inhibited cell growth, migration and epithelial–mesenchymal transition, whereas its knockdown exerted an opposite role. Further analyses showed that circPLEKHM3 sponged miR-9 to regulate the endogenous expression of BRCA1, DNAJB6 and KLF4, and consequently inactivate AKT1 signaling. In addition, AKT inhibitor MK-2206 could block the tumor-promoting effect of circPLEKHM3 depletion, and potentiate Taxol-induced growth inhibition of ovarian cancer cells.

**Conclusions:**

Our findings demonstrated that circPLEKHM3 functions as a tumor suppressor in ovarian cancer cells by targeting the miR-9/BRCA1/DNAJB6/KLF4/AKT1 axis and may be used as a prognostic indicator and therapeutic target in ovarian cancer patients. The new strategy for treating ovarian cancer by a combination therapy of Taxol with MK-2206 is worth further investigation, especially in ovarian cancer patients with loss of circPLEKHM3 expression.

## Introduction

Circular RNAs (circRNAs) are a new class of regulatory non-coding RNA (ncRNA) molecules that form a covalently closed continuous loop structure without a 5′ cap and 3′ poly–A tail [1]. CircRNAs are mainly generated by lasso-driven circularization (exon skipping), intron pairing-driven circularization (direct backsplicing) and RNA-binding proteins [2]. The majority of circRNAs originate from exons of protein-coding genes, and a few are directly cyclized by introns or exon–introns [3]. The improvements in high-throughput RNA sequencing (RNA-seq) and novel bioinformatics tools have led to identifying thousands of circRNAs in various organisms. Because of the covalent loop structure, circRNAs are more stable than linear RNAs and are resistant to exonucleolytic RNA decay [4]. CircRNAs are evolutionarily conserved among organisms, and are abundant in blood [5], saliva [6] and exosomes [7], making them promising diagnostic biomarkers for disease.

Emerging evidence shows that circRNAs may play essential roles in many diseases, including cancer [8]. One of the major functions of circRNAs is to act as miRNA sponges. Several circRNAs were reported to be able to competitively bind to the miRNA response elements with mRNA and long ncRNA to regulate gene expression [9–11]. Dysregulation of the circRNA–miRNA–mRNA axis in signaling pathways is involved in several cancer types. For instance, CDR1as acts as a sponge for miR-7 and miR-671 in the brain, which is essential for maintaining normal brain function [9]. Dysregulation of circHIPK3 changes retinal endothelial cell viability, proliferation, migration, and tube formation by the activation or inhibition of miR-30a-3p [10]. CircCCDC66 functions as a miRNA sponge to protect MYC mRNA from being attacked by miR-33b and miR-93 in colon cancer [11]. In addition, exon–intron circRNAs can influence transcription via interaction with U1 snRNP, Pol II, and the gene promoter [12]. Some circRNAs can act as ‘scaffolding’ for RNA-binding proteins influencing post-transcriptional gene regulation [13]. Although a large number of circRNAs have been identified by RNA-seq, the functions of most circRNAs remain elusive.

Ovarian cancer is the leading cause of death from gynecological malignancy worldwide [14]. More than 75% of affected women are diagnosed at advanced stages with vague and non-specific symptoms. Less than one-third of late-stage patients survive 5 years after diagnosis [15]. Most patients develop metastatic disease after surgery and intensive platinum–taxane chemotherapy. Hence, a major barrier to the treatment of ovarian cancer is identifying novel prognostic biomarkers that can distinguish patients at high risk for relapse and whether any of these biomarkers are potential therapeutic targets. Recent studies have shown that circRNAs play vital roles in the development and progression of many cancers and have promising prognostic and therapeutic potential [7, 16]. However, the expression profiles and underlying molecular mechanisms of circRNAs in ovarian cancer remain largely unknown. Elucidating the role of circRNAs will be critical for understanding the pathogenesis and identifying potential new biomarkers or therapeutic targets for ovarian cancer.

In this study, we compared the expression profiles of circRNAs in ovarian cancer and normal ovarian tissues, and found that a circular RNA derived from PLEKHM3 termed circPLEKHM3 is significantly down-regulated in ovarian cancer. Further analyses indicated patients with lower circPLEKHM3 expression tend to have worse prognosis. Functional studies showed that circPLEKHM3 acts as a competing endogenous RNA (ceRNA) for miR-9 to up-regulate BRCA1, DNAJB6 and KLF4, which subsequently contribute to the activation of the epithelial–mesenchymal transition (EMT), AKT1/P27kip1 and Wnt/β-catenin signaling pathways, and consequently suppress tumorigenesis and progression in ovarian cancer.

## Materials and methods

### Preparation of RNA-seq libraries for detecting circRNAs

This study was reviewed and approved by the Ethnics Committees of Women’s Hospital of Zhejiang University School of Medicine (Hangzhou, China). Five tumor tissues from ovarian cancer patients and five normal ovarian tissues from patients with benign gynaecological diseases were collected. Total RNA was extracted by the Trizol reagent (Invitrogen, Carlsbad, CA, USA). RNA integrity was assessed by the Agilent 2100 Bioanalyzer System (Agilent Technologies, Palo Alto, CA, USA). Before constructing the complementary DNA (cDNA) library, 1 μg total RNA was treated with NEBNext rRNA Depletion Kit (NEB, Ipswich, MA, USA, Cat# E6318) to remove ribosomal RNA (rRNA). Then, strand-specific RNA-seq libraries were prepared using the NEBNext Ultra Directional RNA Library Prep Kit (NEB, Cat# E7420) for Illumina according to the manufacturer’s instructions. The libraries were sequenced with a HiSeq X10 sequencer (Illumina, San Diego, CA, USA) with paired-end reads of length 2 × 150 bp. Approximately, 150 million reads were generated for each sequencing library (Additional file 1: Table S1).

### Identification of circRNAs

The output RNA-Seq sequence reads were pre-processed with Trim Galore (http://www.bioinformatics.babraham.ac.uk/projects/trim_galore/) (Additional file 1: Table S1). Adapters and sequences with low quality (base quality < 20) were removed before the analysis. The trimmed reads were first mapped to human reference genome (hg19) and gene annotation database (Ensembl genes v75 — www.ensembl.org) using TopHat2 (v2.0.13) [17]. Then, all the unmapped reads were used to identify circRNAs by CIRCexplorer2 [18]. The expression level of circRNA was estimated as the ratio of the number of back-spliced junction reads to the maximum number of reads spanning the linear-spliced junction of the same exon(s) in each library. To identify circRNAs expressed independently of their parental genes, only circRNAs whose correlations with their parental genes were not significant (*P*-value > 0.05) were used for subsequent analysis. The expression difference in circRNAs between tumor and normal samples was examined using Students’ *t*-test.

### Real-time quantitative RT-PCR

Total RNA was extracted with Trizol, and 1 μg total RNA was reverse transcribed into cDNA in a reaction volume of 20 μl using PrimeScript™ RT reagent Kit with gDNA Eraser (TAKARA, Shiga, Japan). Primers for real-time quantitative RT-PCR (qRT-PCR) analysis of human circPLEKHM3, linear PLEKHM3, and GAPDH are provided in Additional file 2: Table S2. Before the analysis, total RNA was incubated at 37 °C for 10 min with or without RNase R exonuclease (NEB).

### Western blot analysis and immunohistochemistry

Western blot analysis was performed following the standard protocol as previously described [19]. For immunohistochemistry analysis, the formalin-fixed and paraffin-embedded (FFPE) samples were first deparaffinized and rehydrated, followed by PBS washing. Antigen retrieval was performed in 0.01 M sodium citrate buffer (pH 6.0) at 100 °C for 15 min. The positive cells were scored as: 0 for < 5%, 1 for 6–25%, 2 for 26–50%, 3 for 51–75% and 4 for 76–100%. Staining intensity was scored as: 0 for no staining, 1 for weak, 2 for moderate, and 3 for strong. Immunoreactive scores were calculated by multiplying these two grading scores, which ranged from 0 to 12. The antibodies used are listed in Additional file 2: Table S2.

### Single-molecule RNA in-situ hybridization

The expression of circPLEKHM3 in ovarian cancer tissues was evaluated by BaseScope Assay (Advanced Cell Diagnostics (ACD), Newark, CA, USA). A 1ZZ BaseScope probe targeting the junction sequences of circPLEKHM3 (903-11 nt) was designed, termed as BA-Hs-PLEKHM3-E3-circRNA-Junc (ACD, Cat# 700001). FFPE tissue samples were prepared following the manufacturer’s protocol. BaseScope assays were performed using BaseScope Detection Reagent Kit-RED (ACD, Cat# 322900) in accord with the manufacturer’s protocol. Chromogenic detection was performed using BaseScope Fast RED followed by counterstaining with hematoxylin (American MasterTech Scientific, Lodi, CA). At 20X magnification, the number of visible red dots in 20 randomly scanned regions of each image is used to measure the CircPLEKHM3 expression.

### Cell culture

A2780 cells were cultured in RPMI-1640 media (GIBCO, Australia) supplemented with 10% FBS (GIBCO), penicillin (100 U/ml) and streptomycin (100 ng/ml). OV90 cells were grown in a 1:1 mixture of MCDB 105 medium and medium 199 (GIBCO) supplemented with 15% FBS, penicillin (100 U/ml) and streptomycin (100 ng/ml). MDAH2274 cells were grown in DMEM media (GIBCO) supplemented with 10% FBS, penicillin (100 U/ml) and streptomycin (100 ng/ml). All cells were grown at 37 °C in 5% CO_2_.

### Knockdown of circPLEKHM3

Small interference RNAs (siRNAs) that target the junction sequence of circPLEKHM3 were designed and synthesized by GenePharma (Shanghai, China) (Additional file 2: Table S2). Cells were transfected with these siRNAs with GeneMute™ reagent (SignaGen Laboratories, Rockville, MD, USA). All transfection assays were carried out in triplicate.

### Identification of genes altered by knockdown of circPLEKHM3

mRNA sequencing libraries were prepared for OV90 circPLEKHM3 knockdown and scrambled control cells using the TruSeq RNA Sample Preparation Kit from Illumina as described previously [20]. Sequencing data were preprocessed and mapped as described above. Briefly, the trimmed reads were mapped to human reference genome (hg19) and gene annotation database (Ensembl genes v75) using TopHat2 (v2.0.13) [17] (Additional file 3: Table S3). Transcripts were then constructed and identified using Cufflinks [21]. Differentially expressed genes (DEGs) (adjusted *P*-values < 0.05) were determined by Cuffdiff [21].

### Overexpression of circPLEKHM3

Primers for constructing the pLO–ciR–circPLEKHM3 vector were designed (Additional file 2: Table S2). PLO–ciR vector was purchased from GENESEED (Guangzhou, China). Lentivirus particles were generated in the 293T packaging cells by transfection with the pMD2.G pseudotyping plasmid (Addgene, Teddington, UK, Cat# 12259), the psPAX2 packing plasmid (Addgene, Cat# 12260), and either the PLO–ciR–PLEKHM3, or PLO–ciR viral vector plasmids. Transfections were performed with liptofectamine 3000 in a 6–cm dish.

### CCK8 assay

Cell proliferation was tested with the CCK8 kit (DOJINDO, Kumamoto, Japan, Cat# CK04). Cells were seeded in 96-well plates with approximate 2000 cells/well in 100 μl medium in quintuplicate. CCK8 was added into wells at 0, 24, 48, 72, 96, and 120 h respectively, and incubated for 2.5 h. The absorbance was measured at a wavelength of 450 nm.

### Cell migration assay

The starved cells (10^5^ cells for A2780, 3 × 10^4^ cells for OV90 and MDAH2274) were plated with 300 μL serum-free media into the prepared invasion transwells, and placed above media containing 10% FBS. Plates were incubated in 5% CO_2_ at 37 °C overnight. Images were captured from each membrane and the number of migratory cells was counted under a microscope.

### Preparation of nuclear and cytoplasmic extracts

The nuclear and cytoplasmic fraction of cells was isolated using the PARIS™ kit (Ambion, Austin, TX, USA, Cat# AM1921). About 10^7^ cells were washed with PBS on ice followed by centrifugation at 500×*g* for 5 min. Cell pellets were resuspended in 500 μl cell fraction buffer, incubated on ice for 10 min, and then centrifuged at 500×*g* and 4 °C for 5 min to separate the nuclear and cytoplasmic cell fractions. Nuclear pellets were homogenized with the cell disruption buffer.

### Luciferase report assays

The psiCHECK™-2 Vector (Promega, Madison, WI, USA, Cat# C8021), which contains *Renilla* and firefly luciferase reporter genes, was prepared for luciferase report assays with the full-length circPLEKHM3 or the 3′ UTR of KLF4 and DNAJB6. In the mutant vectors, the miR-9 binding sites in circPLEKHM3 or the 3’UTR of KLF4 and DNAJB6 variant 1 were mutated on psiCHECK™-2 Vectors. The constructed psiCHECK™-2 Vectors were transfected into cancer cells with miRNA mimic and negative control miRNA in 24-well plates. Twenty-four hours after transfection, the cells were lysed with passive lysis buffer (Promega, Cat# E1910), and reporter gene expression was assessed using the Dual Luciferase reporter assay system (Promega, Cat# E1910). The fold change of the relative luciferase activities between miRNA mimic and negative control was calculated.

### RNA fluorescence in-situ hybridization (FISH)

About 6 × 10^4^ cells per well were seeded on cell slides at the bottom of a 24-well plate and incubated. The degree of cell fusion was 80–90% before the experiment. The specific probe to circPLEKHM3 back-splice sequence and miR-9-5p was synthesized by RiboBio (Guangzhou, China) and GeneBio (Shanghai, China), respectively. After cell fixation and permeabilization, cells were treated with prehybridization buffer at 37 °C for 30 min and then hybridized overnight in the hybridization buffer containing 50 μM circPLEKHM3 probe and 250 nM miR-9-5p probe at 37 °C. Subsequently, following the manufacturer’s protocol of RNA FISH (GeneBio), the coverslips were washed with wash buffer I three times, and wash bufferII, wash buffer III and PBS once at 42 °C. After dyeing with DAPI for 10 min, the coverslips were washed with PBS three times. Representative confocal microscopy images of RNA FISH were presented.

### RNA immunoprecipitation

RNA immunoprecipitation (RIP) assay was performed using the Magna RIP™ RNA-Binding Protein Immunoprecipitation Kit (EMD Millipore, Darmstadt, Germany, Cat# 17–700). Briefly, 2 × 10^7^ cells were lysed in 100 μl RIP lysis buffer with the addition of 0.5 μL of protease inhibitor cocktail and 0.25 μL of RNase inhibitor and kept on ice. Then, the lysates were incubated with AGO2 and IgG antibody with protein A/G magnetic beads at 4 °C overnight. The beads were washed three times with wash buffer containing RNase inhibitor and then the RNA was extracted. The abundance of circPLEKHM3 and linear PLEKHM3 was measured by qRT-PCR.

### Animal experiments

Four-week-old female athymic nude mice (BALB/c Nude) were used. Control cells and circPLEKHM3 knockdown cells with pLKO.1 vector (1.2 × 10^6^ cells) were suspended in 0.1 ml RPMI-1640 media without FBS and were subcutaneously inoculated into the flanks of the mice. Six mice were used in each group. Mice were monitored every 5 days for tumor growth, and tumor size was measured using a caliper. Three weeks after inoculation, mice were sacrificed and tumor weight was measured. All experiments were performed in accordance with the Guide for the Care and Use of Laboratory Animals (NIH publication 80–23, revised 1996), with the approval of the Zhejiang University, Hangzhou, China.

### Drug treatment

Cells were seeded in 96-well plates at 5000 cells/well in 100 μl medium, and treated with Taxol (Selleck, Houston, TX, USA, Cat# s1150), MK-2206 (TargetMol, Shanghai, China) or a combination of them for 48 h. The half-life of MK-2206 is about 60 h [22]. Cell viability was determined using a CCK8 assay. All drug treatment assays were carried out in quintuplicate. The index of synergy between MK-2206 and Taxol was evaluated by the *Q*-value in Jin’s formula [23]:

$$ Q={E}_{MK+ Taxol}/\left({E}_{MK}+{E}_{Taxol}-{E}_{MK}\times {E}_{Taxol}\right) $$

where *E*_*MK* + *Taxol*_ is the efficacy of the combination treatment; and *E*_*MK*_ and *E*_*taxol*_ are the efficacies of MK-2206 and Taxol, respectively. In this method, an index > 1.15 indicates synergistic effects between the two treatments; values of the index between 0.85 and 1.15 indicate additive effects of the two treatments; values < 0.85 indicate antagonistic effects of the two treatments. The IC50 values for the treatment of MK-2206 and Taxol were also determined in A2780 scramble and circPLEKHM3 knockdown cells.

### Bioinformatics analysis

Three algorithms (miRanda, Pita and RNAhybrid) were used to predict miR-9 targets [24–26]. The intersection of the three algorithms was considered as the predicted miRNA targets. DEGs with a *q*-value of Student’s *t*-test < 0.01 were subjected to pathway enrichment analysis, using a hypergeometric test based on the KEGG PATHWAY database. To determine if β-catenin signaling is activated upon the knockdown of circPLEKHM3, we used the log2-transformed expression data of CTNNB1 downstream target genes [27] from RNA-seq data of OV90 circPLEKHM3 knockdown cells. The activation of β-catenin signaling was estimated by the PLAGE [28] method, implemented in the R package ‘GSVA’ [29].

### Statistical analysis

The differences between two groups were examined using Student’s *t*-test and a one-way analysis of variance for normally distributed data, and using the Mann–Whitney *U* test for non-normally distributed data. *P-*values < 0.05 were set as the significant threshold. Before the survival analysis, the mean of expression was used to classify patient samples into two groups. The events of overall survival were defined as death, while recurrence-free survival was ended by any disease recurrence or death. A Kaplan–Meier survival analysis was performed to assess the association of circRNA/gene expression with clinical outcome and the *P*-value was calculated with a log-rank test. All statistical analyses were implemented in R statistical packages (https://www.r-project.org/).

## Results

### Down-regulation of circPLEKHM3 is associated with poor prognosis in ovarian cancer

**Fig. 1:**
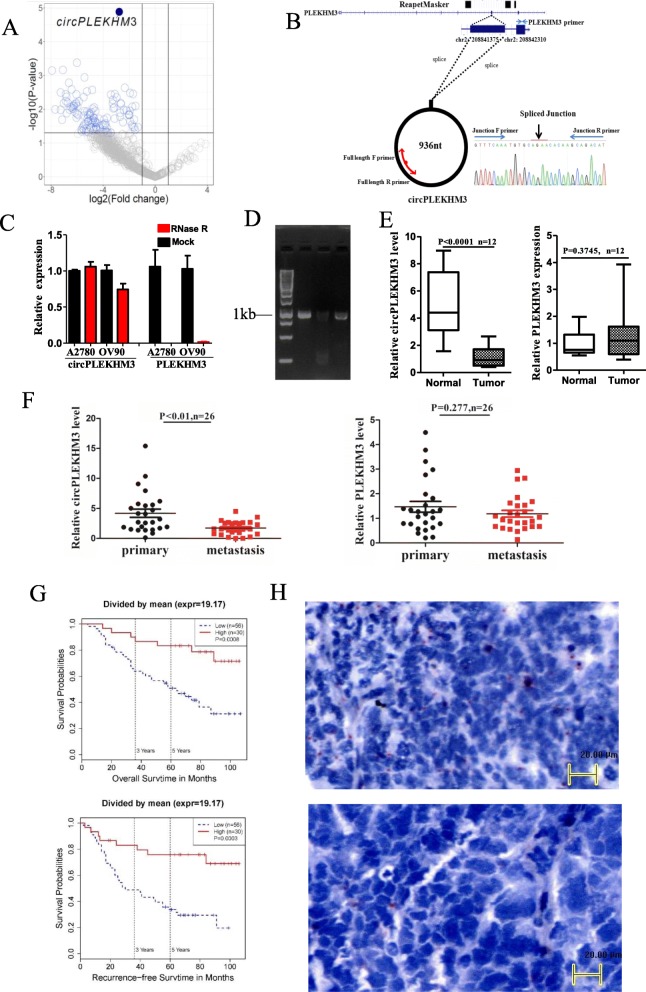
CircPLEKHM3 is down-regulated in ovarian cancer and associated with prognosis. **a** Scatterplot showing the relative expression of circRNA in ovarian tumor and normal tissues. CircPLEKHM3 is marked as a dark circle. **b** Schematic diagram of the generation of circPLEKHM3. CircPLEKHM3 was back-spliced from exon 3 of PLEKHM3. Primers were designed on exon 2 to examine the expression of linear transcripts of PLEKHM3. To validate the existence of circPLEKHM3, primers were designed on the spliced junction, followed by Sanger sequencing. To obtain the full-length sequences of circPLEKHM3, three pairs of primers in opposite directions were designed on exon 3, and their PCR products were sequenced. **c** qRT-PCR analysis of the relative abundance of circPLEKHM3 and PLEKHM3 mRNA in A2780 and OV90 treated with RNase R. **d** PCR product of full-length circPLEKHM3. Primers used to clone full-length circPLEKHM3 are indicated in Fig. 1b and their sequences can be found in Additional file 2: Table S2. **e**, **f** qRT-PCR analysis of the expression of circPLEKHM3 and PLEKHM3 in a new independent cohort including 12 tumor tissues from ovarian cancer patients and 12 normal ovarian tissues from patients with benign gynaecologic diseases (**e**), and 26 primary ovarian carcinoma and matched peritoneal metastatic ovarian carcinomas (**f**). **g** Kaplan–Meier survival analysis of circPLEKHM3 expression in ovarian cancer patients. The expression of circPLEKHM3 was evaluated using a BaseScope assay in 86 FFPE tissues from ovarian cancer patients. **h** Representative images (20× magnification) of the BaseScope assay for circPLEKHM3 in patients with better prognosis (upper) and worse prognosis (lower). CircPLEKHM3 transcript appears as a distinct red dot, with each dot representing a single RNA transcript. Data are presented as mean ± SD; *n* = 3; ** *P* < 0.01, *** *P* < 0.001

To illustrate the role of circRNAs in ovarian cancer, the expression profiles of circRNAs and mRNAs were explored in five ovarian tumor tissues and five normal ovarian tissues using rRNA-depleted RNA sequencing. Volcano plots displayed the log2-fold changes in circRNA expression between ovarian tumor and normal tissues versus its associated -log10 of the *P*-values (Fig. 1a and Additional file 4: Figure S1). CircPLEKHM3 was identified as one of the most significantly down-regulated circRNAs in ovarian tumor tissues. CircPLEKHM3 is spliced from exons of PLEKHM3 on the reverse strand of chromosome 2 (from 208,841,375-208,842,310 bp, hg19 genome build). CircPLEKHM3 was chosen for further investigation because its parental gene PLEKHM3 has not yet been studied in cancer and the PLEKHM3 was not significantly differentially expressed between ovarian tumor and normal tissues. To validate the existence of circPLEKHM3, junction primers were designed (Fig. 1b) to amplify the circPLEKHM3 junction expression in cDNA from A2780 and OV90, followed by Sanger sequencing (Fig. 1b). The sequencing result confirmed the existence of circPLEKHM3, which had an identical junction to that observed in the circBase database (Additional file 5: Figure S2**)**. The circPLEKHM3 junction was only detected in cDNA, whereas its parental gene was detected in both cDNA and genomic DNA from ovarian cancer cell lines (Additional file 6: Figure S3). Furthermore, the expression of circPLEKHM3 was resistant to digestion with RNase R exonuclease, suggesting that the studied RNA species is most likely to be circular in form (Fig. 1c). Finally, three pairs of primers were specifically designed to amplify the full length of circPLEKHM3. Among the three pairs of primers, the forward and reverse primers were next to each other but in the opposite direction (Fig. 1b), so that only circular RNAs could be amplified. PCR results further confirmed the existence of circPLEKHM3 (Fig. 1d). CircPLEKHM3 contained 936 nt and its full-length sequences are given in Additional file 5: Figure S2. Taken together, these experiments validated the existence of circPLEKHM3 that was detected by rRNA-depleted RNA-seq in ovarian tumor tissues.

qRT-PCR assays were performed in independent 12 tumor tissues from ovarian cancer patients and 12 normal ovarian tissues from patients with benign gynaecological diseases. BaseScope assays were also performed in these tissues and additional normal oviduct tissues. Both qRT-PCR and BaseScope assays confirmed that circPLEKHM3 expression was significantly down-regulated in tumor tissues, while PLEKHM3 expression did not differ between tumor and normal tissues (Fig. 1e and Additional file 7: Figure S4). Interestingly, circPLEKHM3 expression was further decreased in peritoneal metastatic ovarian carcinomas as compared with the primary ovarian carcinomas they were derived from, whereas PLEKHM3 expression did not differ between metastatic and primary carcinomas (Fig. 1f and Additional file 8: Figure S5). Furthermore, BaseScope assays were used to assess circPLEKHM3 expression in FFPE tissues from ovarian cancer patients with clinical outcome. Kaplan–Meier survival analysis revealed that down-regulated circPLEKHM3 was associated with short overall survival and recurrence-free survival in cancer patients (Fig. 1g, h). However, the expression of linear PLEKHM3 was not associated with patient outcomes (Additional file 9: Figure S6). These results suggest that circPLEKHM3 is a potential diagnostic and prognostic biomarker and its expression is highly predictive for the clinical outcome in ovarian cancer patients.

### CircPLEKHM3 plays a tumor-suppressive role in ovarian cancer

**Fig. 2:**
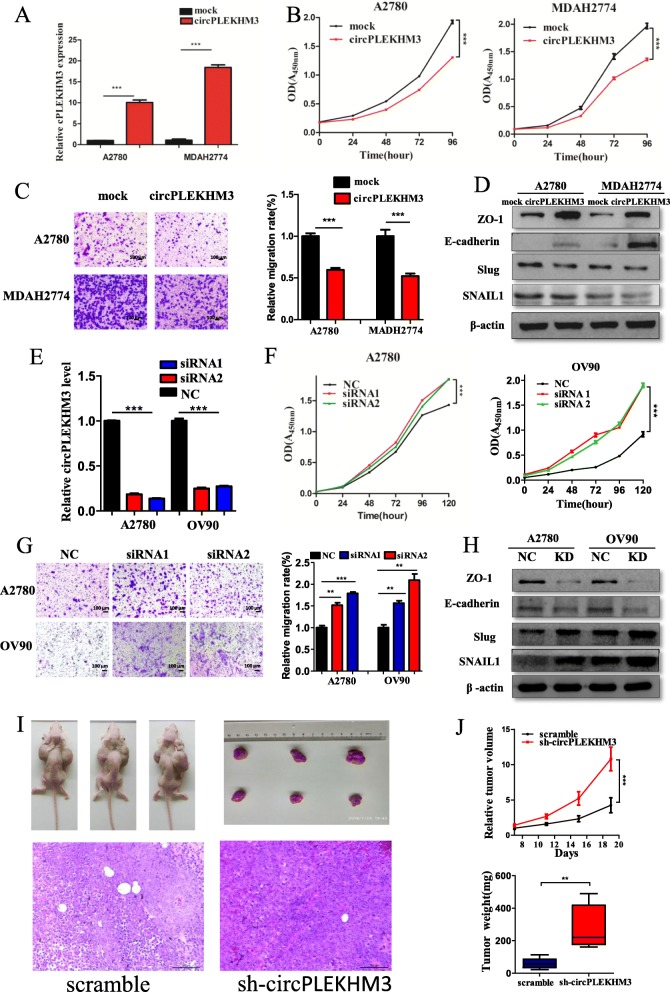
CircPLEKHM3 exerts tumor-suppressive effects in ovarian cancer cells. **a** The relative abundance of circPLEKHM3 in ovarian cancer cells transfected with circPLEKHM3 and mock plasmids determined by qRT-PCR. **b** Proliferation of A2780 and MDAH2274 cells transfected with circPLEKHM3 overexpression and mock vectors assessed with the CCK8 kit. **c** Migration of A2780 and MDAH2274 cells transfected with circPLEKHM3 overexpression and mock vectors measured in a transwell assay at 24 h. **d** Immunoblotting of E-cadherin, ZO-1, Slug and SNAIL1 in A2780 and MDAH2274 cells transfected with circPLEKHM3 overexpression and mock vectors. **e** qRT-PCR analysis of the relative abundance of circPLEKHM3 in OV90 and A2780 cells transfected with two siRNAs and scrambled control. **f** Proliferation of A2780 and MDAH2274 cells transfected with two siRNAs targeting circPLEKHM3 and scrambled control assessed with the CCK8 kit. (**G**) Migration of A2780 and OV90 cells transfected with two siRNAs targeting circPLEKHM3 and scrambled control measured in a transwell assay. **h** Immunoblotting for E-cadherin, ZO-1, Slug and SNAIL1 in A2780 and OV90 cells transfected with siRNA1. **i** Representative images in immunodeficient mice. A2780 scramble and sh-circPLEKHM3 cells were injected subcutaneously in the left and right flank of mice, respectively. **j** The growth curves for tumors and tumor weights of immunodeficient mice injected with A2780 scramble cells and A2780 sh-circPLEKHM3 cells. Tumor volumes were normalized by the first measurement at day 7 after injection. Absolute tumor weights were measured after mice were sacrificed 3 weeks after injection. Six mice per group were used in the nude mouse assay

As described above, circPLEKHM3 expression is significantly down-regulated in cancer tissues and its down-regulation is associated with a poor prognosis in ovarian cancer. These observations suggest that circPLEKHM3 may play a tumor-suppressive role in ovarian cancer. To test this hypothesis, we amplified the full-length cDNA of circPLEKHM3 from A2780 (Additional file 5: Figure S2), and cloned it into the expression vector. CircPLEKHM3 was significantly up-regulated after transfecting the circPLEKHM3 expression vector in A2780 and MDAH2274 cells (Fig. 2a, Additional file 10: Figure S7), whereas the transfection did not affect linear PLEKHM3 mRNA levels (Additional file 11: Figure S8). As a result, the up-regulation of circPLEKHM3 dramatically inhibited cell proliferation (Fig. 2b) and migration (Fig. 2c). Further analysis revealed that the stable overexpression of circPLEKHM3 increased the expression of E-cadherin and zonula occludens (ZO)-1, but decreased the expression of SNAIL1 and Slug (Fig. 2d), indicating that EMT was repressed upon up-regulation of circPLEKHM3. On the contrary, two siRNAs were also designed to target the backsplice junction of circPLEKHM3, which did not affect the expression of its parental gene (Additional file 11: Figure S8). Depletion of circPLEKHM3 (Fig. 2e) promoted ovarian cancer cell proliferation (Fig. 2f), migration (Fig. 2g), and EMT (Fig. 2h). These assays collectively suggested that circPLEKHM3 may act as a tumor suppressor in ovarian cancer. To further confirm the role of circPLEKHM3 in vivo, we established a nude mice xenograft model by subcutaneous inoculation of A2780 cells stably transfected with sh-circPLEKHM3 and scrambled control. We observed that tumor volumes and weights were significantly larger in the sh-circPLEKHM3 than those in the control group (Fig. 2i, j), suggesting that circPLEKHM3 could suppress the tumorigenicity of ovarian cancer cells in vivo. Immunohistochemistry analysis of E-cadherin and SNAIL further indicated that EMT was promoted in immunodeficient mice upon knockdown of circPLEKHM3 (Additional file 12: Figure S9). Taken together, these results suggest that circPLEKHM3 plays a tumor-suppressive role in ovarian cancer in vitro and in vivo.

### CircPLEKHM3 directly binds to miR-9 and suppresses miR-9 activity

**Fig. 3:**
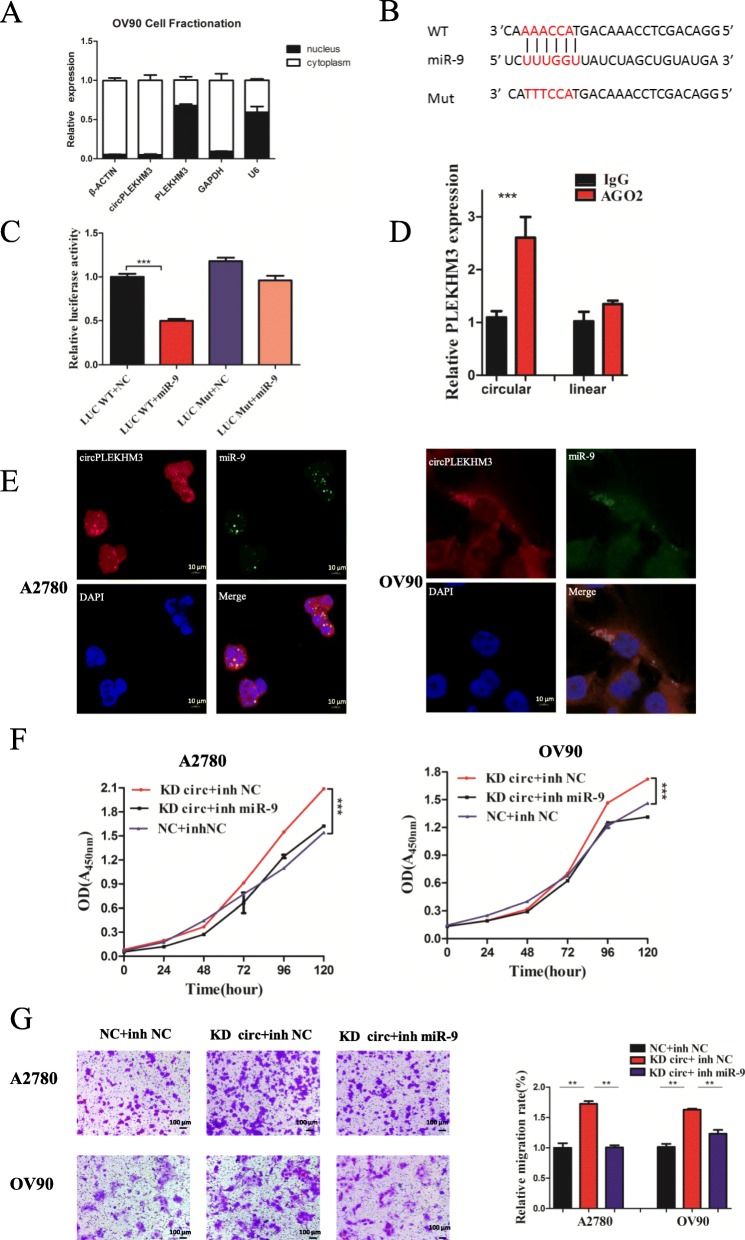
CircPLEKHM3 targets miR-9. **a** qRT-PCR analysis of circPLEKHM3 and PLEKHM3 expression in nuclear and cytosolic fractions of OV90 cells. U6, GAPDH, and β-actin were used for quality control. **b** Luciferase report vector of wild-type (WT) and mutant (Mut) circPLEKHM3. The highlighted sequences represent miR-9 seed sequences or sequences that are complementary to miR-9 seed sequences. In the Mut vector, the miR-9 binding sites in circPLEKHM3 were mutated on psiCHECK™-2 Vectors. **c** Luciferase activity of LUC-circPLEKHM3 wild types and mutants in OV90 cells transfected with miR-9 mimic and negative control mimic. **d** RNA immunoprecipitation assay to measure the amount of circPLEKHM3 and PLEKHM3 pull downed by AGO2 and IgG antibodies in OV90 cells. **e** RNA fluorescence in-situ hybridization for circPLEKHM3 and miR-9 in A2780 and OV90 cells. **f** Cell growth and (**g**) migration assays were performed in circPLEKHM3 knockdown A2780 and OV90 cells transfected with miR-9 inhibitor and negative control (NC). Migration assays were measured 24 h after transfection

CircRNAs have been shown to act as a miRNA sponge to regulate gene expression. CircPLEKHM3 is abundant and stable in the cytoplasm, while PLEKHM3 is abundant in the nucleus (Fig. 3a). We thus investigated the potential miRNAs associated with circPLEKHM3. The full-length sequence of circPLEKHM3 was first blasted in the miRBase database v20 (http://www.mirbase.org/). It was found that circPLEKHM3 possessed a complementary sequence to the miR-9 seed region (Fig. 3b). We then cloned the circPLEKHM3 sequence and the sequence with mutated binding sites of miR-9, and inserted them immediately downstream of the luciferase reporter gene (Additional file 13: Figure S10). To examine whether miR-9 can bind to circPLEKHM3, we co-transfected the miR-9 mimic and luciferase reporters into OV90 cells. Overexpression of miR-9 mimic remarkably reduced luciferase activity to almost 50% when compared with the negative control RNA (Fig. 3c). These results suggest that miR-9 can interact with circPLEKHM3 via the complementary seed region. To further support this finding, we conducted an anti-Ago2 immunoprecipitation assay in OV90, which showed that, compared with IgG, endogenous circPLEKHM3 but not PLEKHM3 was specifically enriched in the immunoprecipitation fraction pulled down by Ago2 (Fig. 3d). Additionally, the co-localization results from FISH analysis confirmed that circPLEKHM3 directly binds to miR-9 (Fig. 3e).

To investigate whether the circPLEKHM3–miR-9 interaction regulates the function of cancer cells, we transfected A2780 and OV90 cells with miR-9 inhibitor after knockdown of circPLEKHM3. Compared with the control, transfection of miR-9 inhibitor significantly rescued cell phenotypes by decreasing cell proliferation and migration (Fig. 3f, g). This suggested that circPLEKHM3 may play a tumor-suppressive role in ovarian cancer cells by suppressing miR-9 activity. Collectively, these results strongly support that circPLEKHM3 binds directly to miR-9 and suppresses miR-9 activity in ovarian cancer cells.

### CircPLEKHM3 suppresses the proliferation and migration of ovarian cancer cells by sponging miR-9 to regulate BRCA1, DNAJB6, and KLF4

Next, three algorithms (miRanda, Pita and RNAhybrid) were used to predict potential targets of miR-9 [24–26]. DNAJB6 and KLF4 were the common targets predicted by all three algorithms. Their expressions were significantly decreased when depleting circPLEKHM3 based on the RNA-seq data from OV90 circPLEKHM3 knockdown cells (Additional file 14: Figure S11). BRCA1 was an experimentally verified target and its gene expression can be regulated by miR-9 [30]. Thus, we only focused on DNAJB6 and KLF4 in subsequent experiments.

**Fig. 4:**
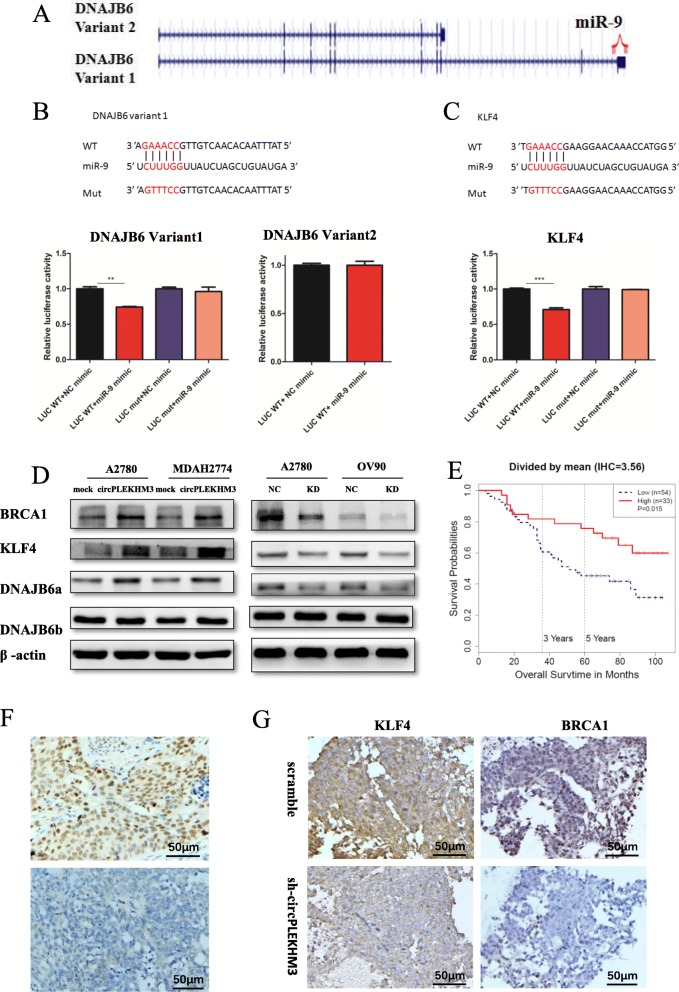
CircPLEKHM3 controls cell proliferation and migration through binding miR-9 to regulate BRCA1, DNAJB6 and KLF4 expression. **a** A schematic illustration of two variants of DNAJB6. The 3’UTR of DNAJB6 variant 2 lacks the miR-9 binding sites. **b** A schematic illustration of the luciferase report vector of DNAJB6 variant 1 3’UTR wild type (WT) and mutant (Mut) (*upper panel*), and the relative luciferase activity of LUC–DNAJB6 variant 1 WT and Mut, and LUC–DNAJB6 variant 2 WT in A2780 cells transfected with miR-9 and control mimics (*lower panel*). The highlighted sequences represent miR-9 seed sequences or sequences that are complementary to miR-9 seed sequences. In the Mut vector, the miR-9 binding sites in the 3’UTR of DNAJB6 variant 1 were mutated on psiCHECK™-2 Vectors. **c** A schematic illustration of the luciferase report vector of KLF4 3’UTR WT and Mut types (*upper panel*), and the relative luciferase activity of LUC–KLF4 WT and Mut in A2780 cells transfected with miR-9 and control mimics (*lower panel*). In the Mut vector, the miR-9 binding sites in the 3’UTR of KLF4 were mutated on psiCHECK™-2 Vectors. **d** Immunoblotting analyses of DNAJB6a, DNAJB6b, KLF4 and BRCA1 in ovarian cancer cells transfected with circPLEKHM3 overexpression, mock vectors, circPLEKHM3 siRNAs and negative control (NC). **e** Kaplan–Meier survival analysis of KLF4 protein expression in ovarian cancer patients. KLF4 protein expression was measured by immunohistochemistry analysis. Patient samples were divided into two groups according to their KLF4 immunohistochemistry scores. **f** Representative images (20× magnification) of immunohistochemical staining for KLF4 in patients with a better prognosis (*upper panel*) and a worse progno (*lower panel*). **g** Representative images (20× magnification) of immunohistochemical staining for KLF4 and BRCA1 in immunodeficient mice injected with A2780 scramble and sh-circPLEKHM3 cells

There are two transcript variants of DNAJB6: variant 1 and 2, according to the UniProtKB database (www.uniprot.org/uniprot/O75190). Variant 1 encodes DNAJB6 protein isoform a (DNAJB6a, 36 kDa), and variant 2 encodes DNAJB6 protein isoform b (DNAJB6b, 26.9 kDa). These two transcript variants have distinct 3′ untranslated regions (UTRs) of DNAJB6 mRNA. Interestingly, the 3′ UTR of variant 1 has a specific sequence complementary to the miR-9 seed region, whereas the 3′ UTR of variant 2 lacks the specific complementary sequence to miR-9 (Fig. 4a). Luciferase reporter assays demonstrated that miR-9 mimic significantly decreased the luciferase activity of DNAJB6 variant 1, while the luciferase activity of variant 2 remained unchanged (Fig. 4b). This verified the interaction between miR-9 and DNAJB6 variant 1 but not variant 2. As expected, knockdown or overexpression of circPLEKHM3 and miR-9 only affected the expression of DNAJB6a, but not of DNAJB6b (Fig. 4d, Additional file 15: Figure S12**)**. The expression of DNAJB6a was analyzed in RNA-seq data from ovarian cancer in The Cancer Genome Atlas (TCGA). Patients with lower DNAJB6a expression tend to have worse prognosis (Additional file 16: Figure S13).

Similar to DNAJB6a, our experiments also confirmed that KLF4 was the target of miR-9 and can be regulated by circPLEKHM3 (Fig. 4c, d, and Additional file 15: Figure S12). KLF4 was reported to play a role in EMT and act as a tumor suppressor in ovarian cancer [31]. Ovarian cancer patients with higher KLF4 expression had consistently better prognoses (Additional file 16: Figure S13). Immunohistochemistry analyses showed that KLF4 was presented in the cytoplasm and nuclear of tumor cells and exhibited stronger staining in tumor tissues from patients with a better prognosis (Fig. 4e, f). KLF4 and BRCA1 exhibited stronger staining in tumor tissues of immunodeficient mice injected with A2780 scramble cells than the mice injected with A2780 sh-circPLEKHM3 cells (Fig. 4g). These results collectively suggest that circPLEKHM3 could act as a ceRNA for miR-9 to regulate the expression of BRCA1, DNAJB6 and KLF4, and suppress the proliferation and migration of ovarian cancer cells.

### CircPLEKHM3 inactivates AKT1 and Wnt/β-catenin signaling pathways

**Fig. 5:**
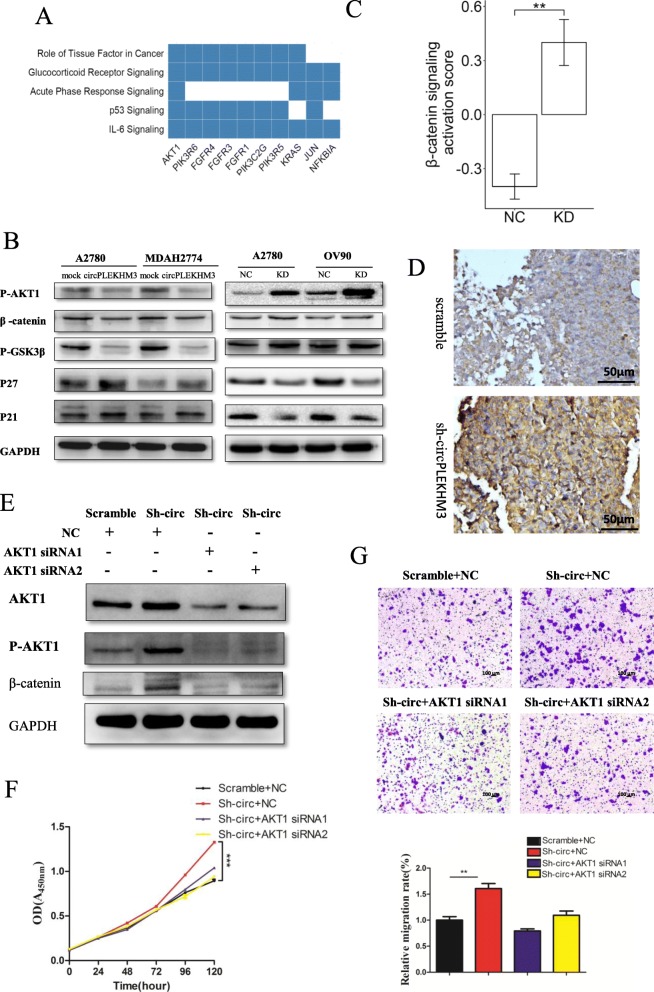
CircPLEKHM3 acts as a tumor suppressor in AKT1 and β-catenin signaling pathways. **a** Top five KEGG pathways enriched for differentially expressed genes upon knockdown of circPLEKHM3 in ovarian cancer cells. Differentially expressed genes were identified by RNA-seq data of circPLEKHM3 knockdown and scrambled control cells, and the 10 most significant genes are shown. **b** Immunoblotting analyses of P-AKT1, β-catenin, P-GSK3β, P21, and P27 in ovarian cancer cells with depletion of circPLEKHM3 or transfected with circPLEKHM3 overexpression and control vectors. **c** β-catenin signaling was activated in OV90 upon knockdown of circPLEKHM3. The pathway activation score was used to estimate the degree to which β-catenin signaling was activated in circPLEKHM3 knockdown and negative control (NC) OV90 cells. **d** Representative images (20× magnification) of immunohistochemical staining for P-AKT1 in immunodeficient mice injected with A2780 scramble and sh-circPLEKHM3 cells. **e** Immunoblotting analyses of AKT1 and P-AKT1 in circPLEKHM3 knockdown A2780 cells transfected with AKT1 siRNAs and negative controls (NC). **f** Cell growth and (**g**) migration assays were performed in circPLEKHM3 knockdown A2780 cells transfected with AKT1 siRNAs and negative controls. Migration assays were measured 24 h after transfection

DNAJB6a and BRCA1 were previously reported to directly negatively regulate the activation of AKT1 [32, 33]. RNA-seq analysis was performed on circPLEKHM3 knockdown and scrambled control cells. DEGs between circPLEKHM3 knockdown and scrambled control cells were detected from these RNA-seq data. Strikingly, AKT1 activation was involved in all the top significant pathways that were enriched for DEGs upon knockdown of circPLEKHM3 (Fig. 5a). Additionally, overexpression of circPLEKHM3 inactivated AKT1, while depletion of circPLEKHM3 increased moderately phosphorylation of AKT1 but had a negligible effect on total AKT1 level in ovarian cancer cells (Fig. 5b, d, e). Involvement of DNAJB6a, KLF4 and AKT1 in Wnt/β-catenin signaling has been reported previously [34–36]. We thus investigated the potential regulatory role of circPLEKHM3 in Wnt/β-catenin signaling. We demonstrated that overexpression of circPLEKHM3 could dephosphorylate GSK3β and decrease β-catenin expression, whereas depletion of circPLEKHM3 showed the opposite effects on GSK3β and β-catenin (Fig. 5b). This is in line with our RNA-seq result that the Wnt/β-catenin signaling pathway was significantly activated upon knockdown of circPLEKHM3 (Fig. 5c). Furthermore, we designed two siRNAs to knock down AKT1 in ovarian cancer cells with depletion of circPLEKHM3. As a result, the upregulation of Wnt/β-catenin that resulted from downregulation of circPLEKHM3 was dramatically attenuated (Fig. 5f); the cell phenotypes were also rescued by decreasing cell proliferation and migration (Fig. 5f, g). Taken together, our data revealed that circPLEKHM3 inactivates the AKT1 and canonical Wnt/β-catenin signaling pathways by regulating the expression of BRCA1, DNAJB6 and KLF4 in ovarian cancer cells.

### AKT inhibitor MK-2206 and Taxol combination therapy exerts synergetic effects on the treatment of ovarian cancer with loss of circPLEKHM3 expression

**Fig. 6:**
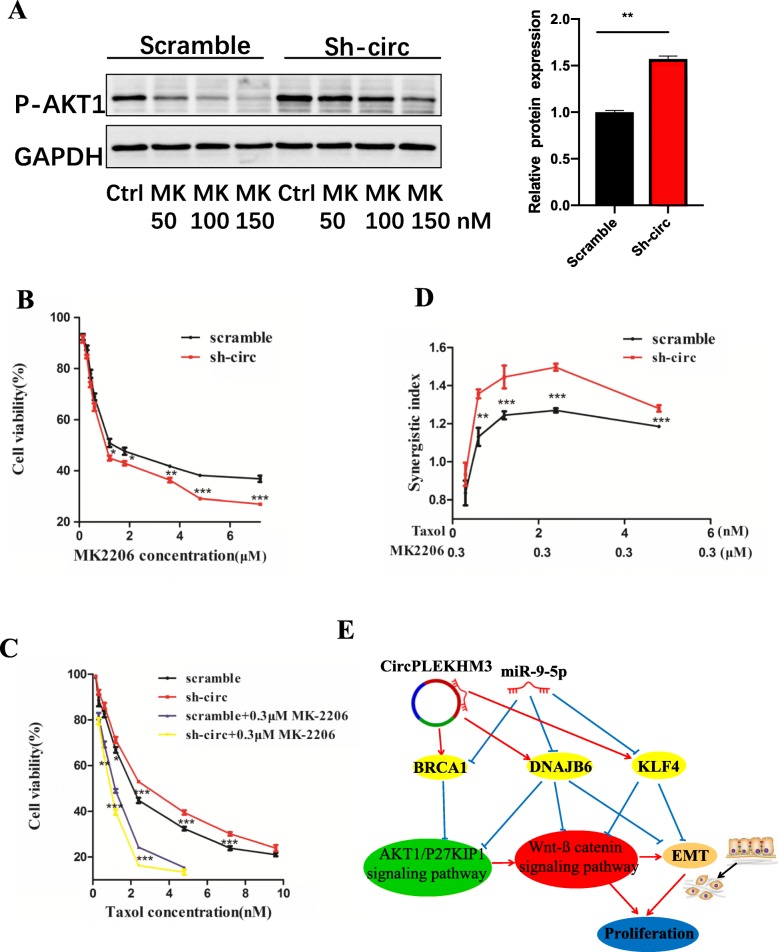
MK-2206 enhances Taxol-induced growth inhibition of ovarian cancer cells, especially in circPLEKHM3 knockdown cells. **a** Immunoblotting analyses of P-AKT1 in A2780 scramble and circPLEKHM3 knockdown cells treated with different concentrations of the AKT1 inhibitor MK-2206 or DMSO for 1 h. **b** Cell viability of A2780 scramble and circPLEKHM3 knockdown cells treated with different concentrations of MK-2206 for 48 h. **c** Cell viability of A2780 scramble and circPLEKHM3 knockdown cells treated with different concentrations of Taxol combined with 0.3 μM MK-2206 for 48 h. **d** The synergistic index of combination treatment of Taxol and MK-2206 in A2780 scramble and circPLEKHM3 knockdown cells. Different concentrations of Taxol (0.3 nM, 0.6 nM, 1.2 nM, 2.4 nM and 4.8 nM) were combined with 0.3 μM MK-2206 in the treatment. **e** Potential molecular mechanisms of circPLEKHM3 in ovarian cancer. CircPLEKHM3 functions as a tumor suppressor in ovarian cancer cells by sponging miR-9 to enhance the endogenous suppressive effect of BRCA1, DNAJB6 and KLF4, and consequently inactivate AKT1 and Wnt-β catenin signaling pathways

Activation of AKT1 and its related signaling pathways is frequently observed in ovarian cancer [37, 38]. Evidence is accumulating that the acquisition of resistance to chemotherapeutic drugs involves the AKT activation pathway [39, 40]. As described above, the down-regulation of circPLEKHM3 could activate AKT1, and thus promote cell proliferation and migration in ovarian cancer. Most patients develop resistance to platinum–taxane chemotherapy in advanced ovarian cancer. MK-2206 is an oral AKT inhibitor that can prevent AKT phosphorylation and enhance antitumor efficacy in combined treatment with standard chemotherapeutic agents or molecular targeted drugs [41]. In this study, we observed that, depending on the concentration, MK-2206 treatment could repress AKT1 phosphorylation in ovarian cancer cells (Fig. 6a). Although the differences in IC50 values of MK2206 between circPLEKHM3 knockdown and scrambled control cells are statistically significant, the effect of circPLEKHM3 knockdown on the MK2206 sensitivity is perceivably small (Fig. 6b and Additional file 3: Table S3). Currently, Taxol is the first-line chemotherapeutic drug for treating ovarian cancer. In contrast to MK-2206, sh-circPLEKHM3 cells were slightly less sensitive to Taxol (Fig. 6c and Additional file 3: Table S3). This is perhaps due to the reduced expression of BRCA1 in circPLEKHM3 knockdown cells that increases the resistance of Taxol for treating ovarian cancer [42]. Overall, MK-2206 enhanced antitumor efficacy in combined treatment with Taxol in both circPLEKHM3 knockdown and scrambled control cells (Fig. 6c). Under MK-2206/Taxol combination therapy, the mean synergetic indexes were 1.1 and 1.3 in scramble and sh-circPLEKHM3cells, respectively (Fig. 6d). This suggests that MK-2206 and Taxol have a synergetic effect on the apoptosis of ovarian cancer cells, and that such synergetic effects are more prominent in ovarian cancer cells with a lower circPLEKHM3 expression. Additionally, the apoptosis analysis further supported these findings **(**Additional file 17: Figure S14). These results collectively demonstrate that although the effect of circPLEKHM3 knockdown on the MK2206 and Taxol sensitivity is small, the MK-2206/Taxol combination therapy exerts a synergetic effect in the treatment of ovarian cancer, and this synergetic effect is increased in ovarian cancer cells with a loss of circPLEKHM3 expression.

## Discussion

Dysregulation of circRNAs plays critical roles in neoplastic initiation and progression. However, the expression and function of circRNAs in ovarian carcinogenesis and progression remains elusive. In this study, we thus performed deep rRNA-depleted RNA sequencing in ovarian cancerous and normal tissues. RNA-seq analysis detected circPLEKHM3 as one of the most significantly down-regulated circular RNAs in ovarian cancer. Interestingly, circPLEKHM3 expression was further decreased in peritoneal metastatic ovarian carcinomas as compared with the primary ovarian carcinomas they were derived from, implying its potential role in ovarian cancer metastasis. CircPLEKHM3 is a novel circRNA and its expression and functional role in cancers has not yet been explored. To evaluate its clinical value, we quantified the expression of circPLEKHM3 in FFPE tissues from ovarian cancer using a BaseScope assay. The BaseScope assay is a new RNA in-situ hybridization technique and has the advantage of effectively preventing non-specific binding of the probe while reducing background interference [43]. The BaseScope assay revealed that ovarian cancer patients with a lower circPLEKHM3 expression tend to have a worse prognosis.

Functionally, the down-regulation of circPLEKHM3 could promote the proliferation and migration of ovarian cancer cells, and induce EMT to facilitate tumor metastasis, whereas the up-regulation of circPLEKHM3 could exert an opposite role. Recent studies have revealed that circRNAs have an important function in gene regulation. For example, circRNAs can function as a miRNA sponge [10, 11], transcription regulator [12], and protein decoy [44], and regulate gene expression at transcriptional or post-transcriptional level. Our results demonstrated that circPLEKHM3 can directly bind to miR-9, and function as a ceRNA to enhance the suppressive effect of miR-9 target genes such as BRCA1, DNAJB6 and KLF4, and consequently inactivate AKT1. The direct interaction between circPLEKHM3 and miR-9 was very evident under the confocal microscope at 60× magnification. Consistently, luciferase and RIP assays further supported that circPLEKHM3 and miR-9 physically bind to each other (Fig. 3e).

RNA-seq analysis of circPLEKHM3 knockdown cells revealed that AKT1 activation is one of the top targeted pathways upon deletion of circPLEKHM3 in ovarian cancer cells. Previous studies have reported that wild-type BRCA1 can negatively regulate AKT1 activation [32, 45]. DNAJB6a can bind to the phosphorylation sites threonine 308 (T308) and serine 473 (S473) of AKT1, inhibiting AKT1 activation [33], although this signaling pathway may need further investigation in ovarian cancer cells. These data are in good agreement with our observations in ovarian cancer. Additionally, KLF4 directly interacts with the C-terminal transactivation domain of β-catenin and inhibits p300/cbp recruitment by β-catenin [35, 46]. β-Catenin also contributes to the transcriptional regulation of AKT1 [47]. Importantly, cell phenotypes were rescued when inhibiting the miR-9 in circPLEKHM3 knockdown cells, implying miR-9 as an essential regulator in this model. Furthermore, when AKT1 was knocked down in ovarian cancer cells with depletion of circPLEKHM3, the upregulation of Wnt/β-catenin that resulted from downregulation of circPLEKHM3 was dramatically attenuated; the cell phenotypes were also rescued by decreasing cell proliferation and migration. Taken together, these data suggested the circPLEKHM3-miR-9 axis is an important regulator in mediating the crosstalk between Wnt/β-catenin and AKT1 signaling pathways that promote the progression of ovarian cancer.

As described above, circPLEKHM3 could hypoactivate AKT1. Our study found that circPLEKHM3 is commonly down-regulated in ovarian cancer. This is consistent with the abnormal activation of AKT1 frequently observed in ovarian cancer [37, 38]. These results provide a rationale for the combination therapy of cisplatin-based chemotherapy drugs with an AKT inhibitor in ovarian cancer. As expected, circPLEKHM3 knockdown cells were more sensitive to AKT inhibitor MK-2206. Moreover, the chemotherapy drug Taxol synergistically improved the effect of MK-2206 in ovarian cancer cells with a loss of circPLEKHM3 expression. Summarizing, circPLEKHM3 has strong potential as a therapeutic target for ovarian cancer. MK2206 is now in phase II clinical trials [48]. Therefore, our results have the potential to translate into effective therapies for ovarian cancer.

Lastly, one caveat should be acknowledged in the study. CircPLEKHM3 was significantly down-regulated in ovarian cancer tissues compared with normal tissues. Its expression was further decreased in peritoneal metastatic ovarian carcinomas compared to primary ovarian carcinomas. To evaluate its potential clinical value, we measured the expression of circPLEKHM3 in FFPE tissues from ovarian cancer using the BaseScope assay. Interestingly, we observed that tumor tissues from patients with short-term survival tend to have fewer positive staining cells than those with long-term survival. This implied that the reduction of circPLEKHM3 expression at tumor tissue level is likely attributed to the reduced number of cells expressing circPLEKHM3. However, the BaseScope assay is a semi-quantitative approach to detect RNA expression using in situ hybridization. There is a detection limit in the BaseScope assay. Some regions without visible dots could be due to extremely low expression of circPLEKHM3. Additionally, we performed single cell cloning of an ovarian cancer cell line (A2780) and found that circPLEKHM3 has a considerable variability in expression among these clones (Additional file 10: Figure S7B). Therefore, due to the technical limitation and large variability in circPLEKHM3 expression, at the current stage we could not completely exclude another potential mechanism that the reduction of circPLEKHM3 expression at tumor tissue level is perhaps attributed to the decreased expression level per cell of circPLEKHM3. Whether the expression level per cell of circPLEKHM3 is reduced or the number of cells expressing circPLEKHM3 is reduced? It is an important question that could be also asked about most studies with qRT-PCR and RNA-seq. Further investigations are required to answer this question. It is worth noting that the reduction in the expression of circPLEKHM3 within tumor tissues that is caused by either of the above two mechanisms (reduction in per cell or number of cells) will likely have the same effect on the expression level of free miR-9 in general in tumor cell populations (also saying, at tumor tissue level). That is, either of the two mechanisms will lead to release more sponged miR-9 into tumor tissues, which subsequently reduces the endogenous suppressive effect of BRCA1, DNAJB6 and KLF4.

## Conclusions

CircPLEKHM3 is down-regulated in ovarian cancer. Patients with a lower circPLEKHM3 expression have a worse prognosis. CircPLEKHM3 was found to act as a tumor suppressor that inhibits cell proliferation. It’s down-regulation promotes EMT to facilitate tumor progression, and induces resistance to the chemotherapy drug Taxol in ovarian cancer. Mechanistically, circPLEKHM3 binds to miR-9 to enhance the endogenous suppressive effect of BRCA1, DNAJB6 and KLF4. The circPLEKHM3-miR-9 axis is an important regulator in mediating the crosstalk between Wnt/β-catenin and AKT1 signaling pathways that promote the progression of ovarian cancer. The combination of Taxol with MK-2206 displays a synergistic effect in cells with circPLEKHM3 loss. These results indicate that circPLEKHM3 is a valuable prognostic biomarker and a promising target for anti-cancer therapy in ovarian cancer. The new strategy of treating ovarian cancer with a combination therapy of Taxol and MK-2206 is worth further consideration, especially in ovarian cancer patients with a loss of the expression of circPLEKHM3.

## Supplementary information

**Additional file 1:** **Table S1.** Quality control metrics of rRNA-depleted RNA sequencing libraries.

**Additional file 2:** **Table S2.** Information on antibodies and primers used in this study.

**Additional file 3:** **Table S3.** IC50 values for the treatment of MK-2206 and Taxol in A2780 scramble and circPLEKHM3 knockdown cells.

**Additional file 4:** **Figure S1.** qRT-PCR analysis of the relative abundance of circPLEKHM3 and PLEKHM3 mRNA in RNA-seq samples. Five tumor tissues from ovarian cancer patients and five normal ovarian tissues from patients with benign gynaecological diseases were used in RNA-seq experiments.

**Additional file 5:** **Figure S2.** Genomic information on circPLEKHM3. (**A**) CircBase annotation for circPLEKHM3 (ID: hsa_circ_0001095). (**B**) Sequences of full-length circPLEKHM3 from Sanger sequencing.

**Additional file 6:** **Figure S3.** PLEKHM3 and circPLEKHM3 detected by agarose gel electrophoresis of A2780 and OV90 cells.

**Additional file 7:** **Figure S4.** BaseScope assay for circPLEKHM3 in normal oviduct, normal ovary and ovarian tumor tissues.

**Additional file 8:** **Figure S5.** BaseScope assay for circPLEKHM3 in primary ovarian carcinoma and matched peritoneal metastatic ovarian carcinomas.

**Additional file 9:** **Figure S6.** PLEKHM3 expression is not associated with survivals of ovarian cancer patients. (**A**) The protein expression of PLEKHM3 was measured by IHC analysis. Nine of eighty-six patients either failed to have a good IHC staining or did not acquired additional FFPE tissue blocks. (**B**) Kaplan–Meier survival analysis of PLEKHM3 expression in ovarian cancer patients. Differences in the survival risk between the two groups were assessed by the Mantel–Haenszel log-rank test.

**Additional file 10:** **Figure S7.** Expression of circPLEKHM3 in ovarian cancer cells. (**A**) The relative expression of circPLEKHM3 in TOV112D, OVCAR-3, HO8910, MDAH2774, OV90, A2780, and IOSE80 cell lines by real time quantitative RT-PCR. (**B**) Expression of circPLEKHM3 in single cell clones from A2780 cells.

**Additional file 11:** **Figure S8.** The relative expression of PLEKHM3 after knockdown or overexpression of circPLEKHM3 in ovarian cancer cells by real time quantitative RT-PCR.

**Additional file 12:** **Figure S9.** Representative images (20× magnification) of immunohistochemical staining for E-cadherin and SNAIL in immunodeficient mice injected with A2780 scramble and shcircPLEKHM3 cells.

**Additional file 13:** **Figure S10.** Sanger sequencing of luciferase report vectors of circPLEKHM3, DNAJB6 variant 1 and KLF4 3′ UTRs. The highlighted sequences represent parts of miR-9 seed sequences that were mutated on psiCHECK™-2 Vectors.

**Additional file 14:** **Figure S11.** The relative expression of KLF4 and DNAJB6 after knockdown of circPLEKHM3 in OV90 cells. The expression of KLF4 and DNAJB6 was quantified by FPKM (fragments per kilobase of exon model per million reads mapped) in the RNA-seq data from OV90 circPLEKHM3 knockdown and negative control (NC) cells.

**Additional file 15:** **Figure S12.** The relative expressions of DNAJB6a (DNAJB6 isoform a), DNAJB6b (DNAJB6 isoform b), KLF4 and BRCA1 in A2780 cells transfected with miR-9 mimic and inhibitor by immunoblotting analysis.

**Additional file 16:** **Figure S13.** Ovarian cancer patients with a higher expression of DNAJB6 variant 1 and KLF4 are associated with better prognoses. The expression of DNAJB6 variant 1 was retrieved from RNA-seq data of ovarian cancer in TCGA. The expression of KLF4 was downloaded from the Gene Expression Omnibus (GSE3149). Differences in the survival risk between the two groups were assessed by the Mantel–Haenszel log-rank test.

**Additional file 17:** **Figure S14.** Apoptosis assays of cells with treatment Taxol and/or MK2206. Cells were treated with Taxol (3 nM) alone, MK2206 (3 μM) alone or Taxol in combination with MK-2206. Cells were harvested and stained using the Annexin V-FITC apoptosis detection kit after about 48 h of treatment. Cells with Annexin V+ staining located in the right upper and lower quadrants were considered as apoptotic cells (mean ± SEM, n = 3).

## Data Availability

The raw sequence data will be deposited in the NCBI short read archive (SRA) upon acceptance of the data for publication. All other data that support the findings of this study are available from the corresponding authors upon reasonable request.

## Abbreviations

ceRNA: competing endogenous RNA
circRNAs: Circular RNAs
DEGs: Differentially expressed genes
DNAJB6a: DNAJB6 protein isoform a
DNAJB6b: DNAJB6 protein isoform b
EMT: Epithelial-mesenchymal transition
FFPE: Formalin-fixed and paraffin-embedded
FISH: RNA fluorescence in-situ hybridization
ncRNA: non-coding RNA
qRT-PCR: quantitative real-time PCR
RIP: RNA immunoprecipitation
RNA-seq: RNA sequencing
rRNA: ribosomal RNA
TCGA: The Cancer Genome Atlas
UTRs: Untranslated regions
