# Supplementary material for: CircPLEKHM3 acts as a tumor suppressor through regulation of the miR-9/BRCA1/DNAJB6/KLF4/AKT1 axis in ovarian cancer
Source: Mol Cancer. 2019 Oct 17;18:144. doi: 10.1186/s12943-019-1080-5 (PMC6796346; doi:10.1186/s12943-019-1080-5)
Supplement: Supplementary file 1 — Additional file 1: Table S1. Quality control metrics of rRNA-depleted RNA sequencing libraries. [file 12943_2019_1080_MOESM1_ESM.pdf]

Table S1. Quality control metrics of RNA sequencing libraries.

Sample ID	Treatment	Tissue	Yield (Gb)	# Reads	% of >= Q30 Bases (PF)	Mean Quality Score (PF)	% of mapping
OC35	Rnase_R+	Tumor	19.0	133,516,188	96.0%	37.7	82.3
OC55	Rnase_R+	Tumor	13.1	92,270,002	92.1%	36.4	78.4
OC2	Rnase_R+	Tumor	11.2	77,256,646	97.4%	39.0	86.9
OC10	Rnase_R+	Tumor	18.7	128,177,434	96.2%	38.4	78.9
OC30	Rnase_R+	Tumor	18.4	129,730,690	96.2%	38.0	78.2
NC6	Rnase_R+	Normal	19.5	134,536,734	96.3%	38.5	83.3
NC4	Rnase_R+	Normal	19.8	136,112,844	96.4%	38.6	85.4
NC14	Rnase_R+	Normal	22.0	156,582,656	96.1%	38.5	71.7
NC52	Rnase_R+	Normal	18.9	130,031,908	95.9%	37.9	83.4
NC54	Rnase_R+	Normal	19.0	131,241,188	96.8%	38.3	85.9
