# Supplementary material for: CircPLEKHM3 acts as a tumor suppressor through regulation of the miR-9/BRCA1/DNAJB6/KLF4/AKT1 axis in ovarian cancer
Source: Mol Cancer. 2019 Oct 17;18:144. doi: 10.1186/s12943-019-1080-5 (PMC6796346; doi:10.1186/s12943-019-1080-5)
Supplement: Supplementary file 2 — Additional file 2: Table S2. Information on antibodies and primers used in this study. [file 12943_2019_1080_MOESM2_ESM.pdf]

Table S2. Information on antibody and primers used in this study.

Antibody target	Vendor	Cat#
P-GSK-3β	Diagbio	db1715
BRCA1	Santa Cruz	6954
KLF4	Diagbio	db71
DNAJB6	Diagbio	db2997
P-AKT1	Diagbio	db1727
P21	Cell Signaling Technology	12D1
P27	Diagbio	db3374
ZO-1	Abcam	D6L1E
N-cadherin	Abcam	D4R1H
E-cadherin	Abcam	24E10
Snail	Abcam	C15D3
Vimentin	Abcam	D21H3
β-actin	Abcam	13E5
GAPDH	Diagbio	db106
β-catenin	Cell Signaling Technology	D10A8

Target	Primers/siRNAs	Sequence(5'→3')
circPLEKHM3	Forward primer	CTCAGGAAGAACAAACGCCA
	Reverse primer	CAGCTTTGCCAGTAACTGTCA
linear PLEKHM3	Forward primer	CAGCCTTAGAAGTTACGGAGG
	Reverse primer	ACATTTCTCATAGCACCATTGTCTG
full length circPLEKHM3	Forward primer	GGCCATCATGATGACTATACACAG
	Reverse primer	AAGCCCTGGTGAGATTGTCAGCTC
full length circPLEKHM3	Forward primer	TGCTGTGCCCGGTTACATGGGGCGGC
	Reverse primer	GCATGCACTACTTCCCAAAGCTTCTG
full length circPLEKHM3	Forward primer	CCACCGTCCCCTGTCCTGGAC
	Reverse primer	CAGCAGCCCACTGGTTTTCTTCTG
β-actin	Forward primer	GGCACCACACCTTCTACAAT
	Reverse primer	GCCTGGATAGCAACGTACAT
GAPDH	Forward primer	AGCTCACTGGCATGGCCTTC
	Reverse primer	CGCCTGCTTCACCACCTTCT
circPLEKHM3	siRNA1	CAAAUGUGCAGAACACAAGdTdT
	siRNA2	GUGCAGAACACAAGCAGACdTdT
pLO-ciR-circPLEKHM3	overexpression vector	CGGAATTCTGAAATATGCTATCTTACAG<br>AACACAAGCAGACATTCCCAA,<br>GGAATTCCATATGTCAAGAAAAAATATA<br>TTCACCTGCACATTTGAAACTCTGAGC
