# Supplementary material for: CircPLEKHM3 acts as a tumor suppressor through regulation of the miR-9/BRCA1/DNAJB6/KLF4/AKT1 axis in ovarian cancer
Source: Mol Cancer. 2019 Oct 17;18:144. doi: 10.1186/s12943-019-1080-5 (PMC6796346; doi:10.1186/s12943-019-1080-5)
Supplement: Supplementary file 3 — Additional file 3: Table S3. IC50 values for the treatment of MK-2206 and Taxol in A2780 scramble and circPLEKHM3 knockdown cells. [file 12943_2019_1080_MOESM3_ESM.pdf]

Table S3. IC50 values for the treatment of MK-2206 and Taxol in A2780 scramble and circPLEKHM3 knockdown cells.

Cell line	Treatment sequence	IC50 (Taxol: nM; MK2206:μM)			
		Single		Combination	
		TA	MK	TA	MK
Scramble	TA MK	2.320	2.176	1.098	0.300
Sh-circ	TA MK	3.023	1.490	0.862	0.300
