# Supplementary material for: CircPLEKHM3 acts as a tumor suppressor through regulation of the miR-9/BRCA1/DNAJB6/KLF4/AKT1 axis in ovarian cancer
Source: Mol Cancer. 2019 Oct 17;18:144. doi: 10.1186/s12943-019-1080-5 (PMC6796346; doi:10.1186/s12943-019-1080-5)
Supplement: Supplementary file 4 — Additional file 4: Figure S1. qRT-PCR analysis of the relative abundance of circPLEKHM3 and PLEKHM3 mRNA in RNA-seq samples. Five tumor tissues from ovarian cancer patients and five normal ovarian tissues from patients with benign gynaecological diseases were used in RNA-seq experiments. [file 12943_2019_1080_MOESM4_ESM.pdf]

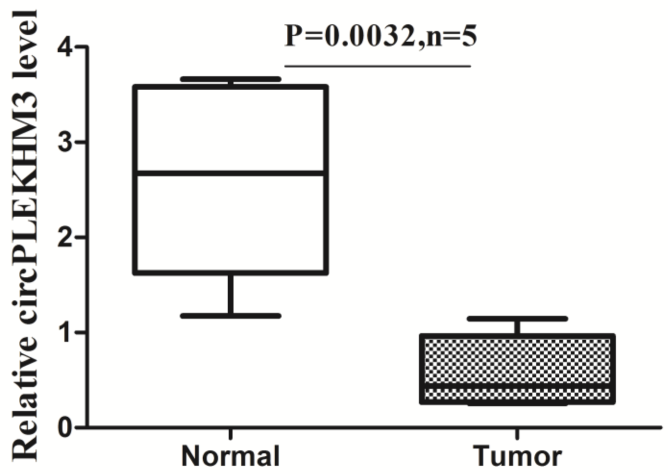

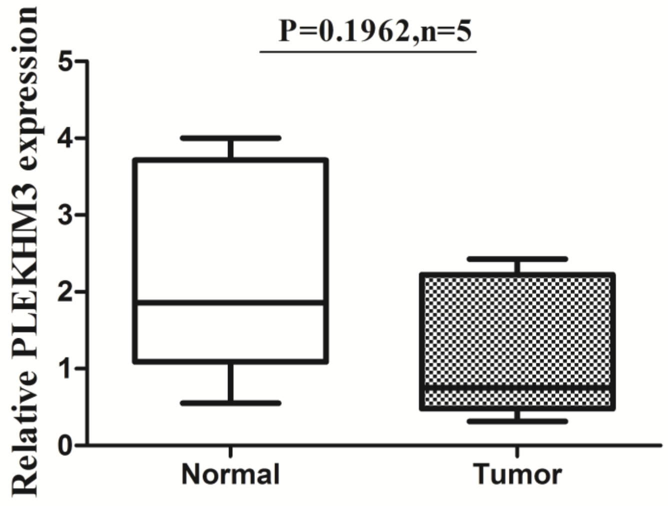

Figure S1. qRT-PCR analysis of the relative abundance of circPLEKHM3 and PLEKHM3 mRNA in RNA-seq samples. Five tumor tissues from ovarian cancer patients and five normal ovarian tissues from patients with benign gynaecological diseases were used in RNA-seq experiments.
