# Supplementary material for: CircPLEKHM3 acts as a tumor suppressor through regulation of the miR-9/BRCA1/DNAJB6/KLF4/AKT1 axis in ovarian cancer
Source: Mol Cancer. 2019 Oct 17;18:144. doi: 10.1186/s12943-019-1080-5 (PMC6796346; doi:10.1186/s12943-019-1080-5)
Supplement: Supplementary file 5 — Additional file 5: Figure S2. Genomic information on circPLEKHM3. (A) CircBase annotation for circPLEKHM3 (ID: hsa_circ_0001095). (B) Sequences of full-length circPLEKHM3 from Sanger sequencing. [file 12943_2019_1080_MOESM5_ESM.pdf]

A

ID	hsa_circ_0001095
Alias	hsa_circ_000023
Position	chr2:208841374-208842310
Strand	-
Genomic length	936
Spliced sequence length	936
Annotation	ANNOTATED<br>CDS<br>coding<br>INTERNAL<br>OVCODE<br>OVEXON
Repeats	None
Best transcript	NM_001080475
Gene symbol	PLEKHM3
Experimental exidence	Memczak2013

B

AACACAAGCAGACATTCCCAAACATTCTAAAGAAGGGTTACCTG
GAGATTAGAAAGGACCATGACAGTTACTGGCAAAGCTGTTATGCA
GAACTTTCACCTTACAACTTATACTTCTACAGCCTCGACAGCAGTG
GGAATCAAAACCTTTATGCCACGTACCAGCTTTCACACTTCCAGA
GCATATCTGTTTTAGGCAACCTGGAGGCCAGGATGGTGGATACTG
TTTTGTATGACAACACTCAGCTACAGCTAAAGGCAGAGTCACCAT
GGGAGGCTTTGGACTGGGGACAGAAGCTTTGGGAAGTAGTGCAT
GCTGCTGTGCCCGGTTACATGGGGCGGCAGAATGAGCTGACAATC
TCACCAGGGCTTGGCCATCATGATGACTATACACAGAATCATAGTT
TCCAGAAGAAAACCAGTGGGCTGCTGCCACCGTCCCCTGTCCTG
GACAGCTCCAAACAGTACCAAAACATCCTCAAATCAGGGACTCT
CTACAGGCTGACTGTCCAAAACAACTGGAAGGCATTTACATTTGT
GCTGAGCAGGGCTTACCTTATGGCTTTTCAGCCTGGCAAGCTAGA
CGAGGATCCACTGTTGAGCTACAACGTGGACGTGTGTCTGGCTGT
CCAGATGGACAACCTGGATGGCTGCGACTCTTGCTTTCAAGTCAT
TTTCCCCCAGGATGTCCTTCGCCTCCGAGCTGAGACCCGACAGAG
GGCTCAGGAATGGATGGAGGCTCTGAAGATAGCTGCCAATGTGGC
GAGGAGTTCAGAGCAAAACCTGCAAGTCACACTGAGGAACAAA
CCCAAGGATCAAATGGGTGGGCATGAACTCAGGAAGAACAAACG
CCAATCTGTGACTACCAGCTTCCTGAGCATTTTGACGACTTTGTCT
TTGGAACGAGGACTCACTGCTCAGAGTTTCAAATGTGCAG

Figure S2. Genomic information on circPLEKHM3.
(A) circBase annotation for circPLEKHM3 (ID: hsa_circ_0001095). (B) Sequence of full-length circPLEKHM3 from Sanger sequencing.
