# Supplementary figures and images for: CircPLEKHM3 acts as a tumor suppressor through regulation of the miR-9/BRCA1/DNAJB6/KLF4/AKT1 axis in ovarian cancer
Source: Mol Cancer. 2019 Oct 17;18:144. doi: 10.1186/s12943-019-1080-5 (PMC6796346; doi:10.1186/s12943-019-1080-5)

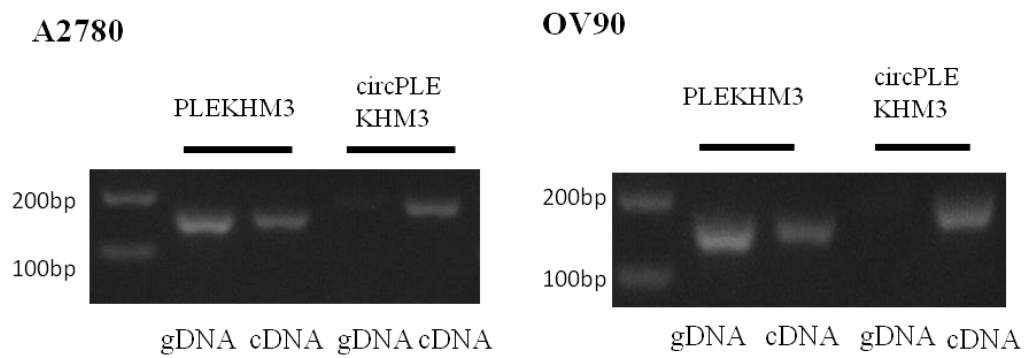

**Figure S3.** PLEKHM3 and circPLEKHM3 detected by Agarose gel electrophoresis in A2780 and OV90 cells.

Supplement: Supplementary file 6 — Additional file 6: Figure S3. PLEKHM3 and circPLEKHM3 detected by agarose gel electrophoresis of A2780 and OV90 cells. [file 12943_2019_1080_MOESM6_ESM.pdf]
