# Supplementary material for: CircPLEKHM3 acts as a tumor suppressor through regulation of the miR-9/BRCA1/DNAJB6/KLF4/AKT1 axis in ovarian cancer
Source: Mol Cancer. 2019 Oct 17;18:144. doi: 10.1186/s12943-019-1080-5 (PMC6796346; doi:10.1186/s12943-019-1080-5)
Supplement: Supplementary file 7 — Additional file 7: Figure S4. BaseScope assay for circPLEKHM3 in normal oviduct, normal ovary and ovarian tumor tissues. [file 12943_2019_1080_MOESM7_ESM.pdf]

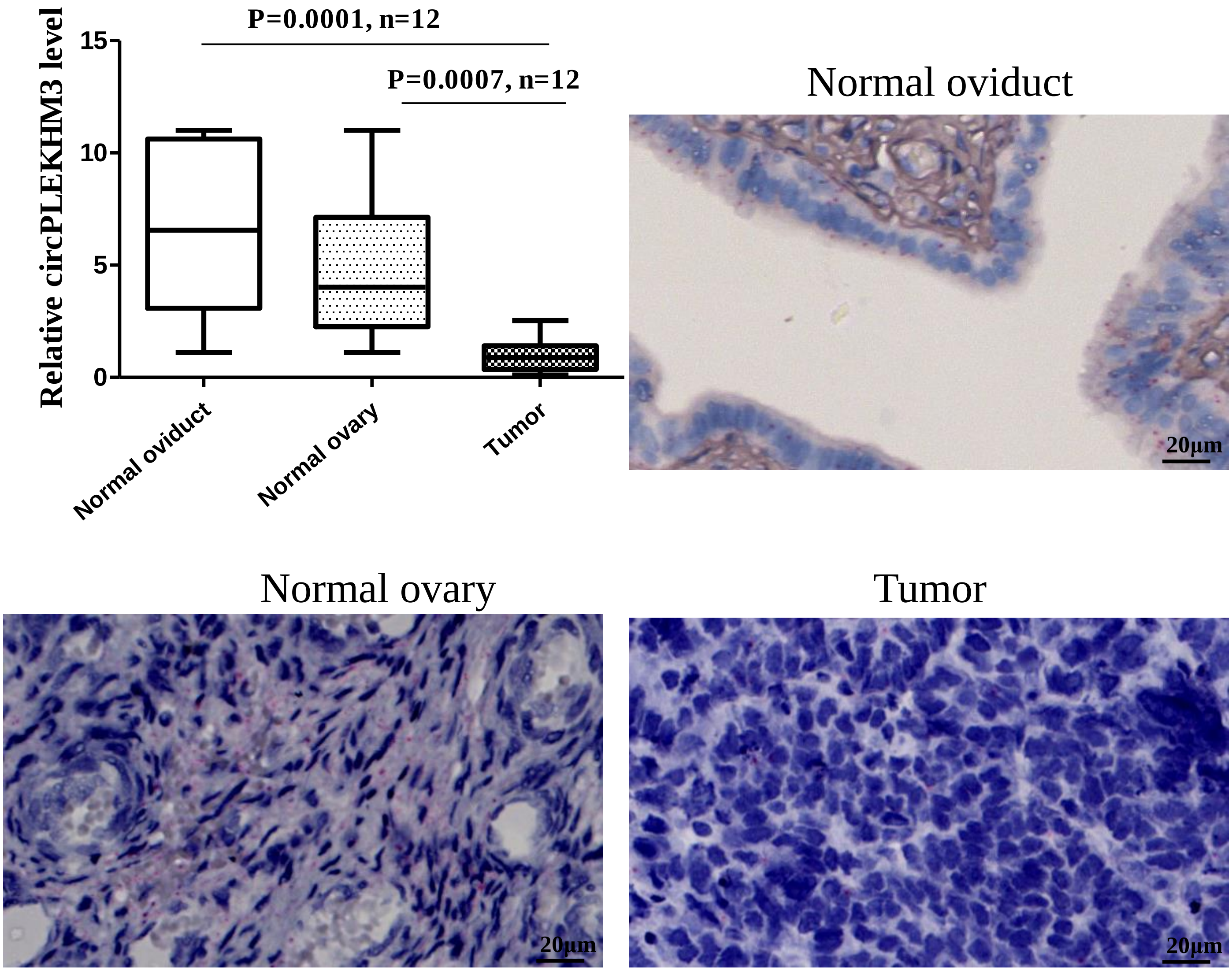


**Figure S4.** BaseScope assay for circPLEKHM3 in normal oviduct, normal ovary and ovarian tumor tissues.
