# Supplementary material for: CircPLEKHM3 acts as a tumor suppressor through regulation of the miR-9/BRCA1/DNAJB6/KLF4/AKT1 axis in ovarian cancer
Source: Mol Cancer. 2019 Oct 17;18:144. doi: 10.1186/s12943-019-1080-5 (PMC6796346; doi:10.1186/s12943-019-1080-5)
Supplement: Supplementary file 8 — Additional file 8: Figure S5. BaseScope assay for circPLEKHM3 in primary ovarian carcinoma and matched peritoneal metastatic ovarian carcinomas. [file 12943_2019_1080_MOESM8_ESM.pdf]

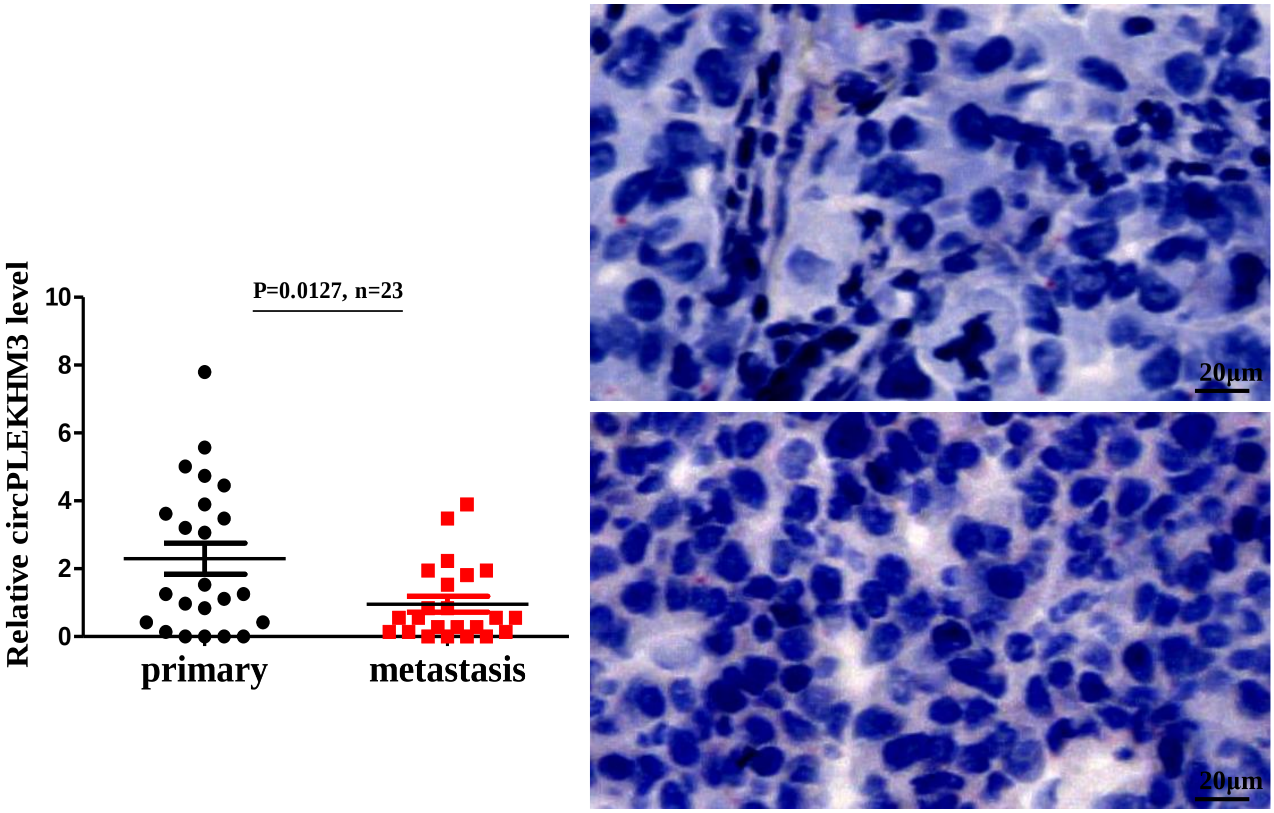

Figure S5. BaseScope assay for circPLEKHM3 in primary ovarian carcinoma and matched peritoneal metastatic ovarian carcinomas.
