# Supplementary material for: CircPLEKHM3 acts as a tumor suppressor through regulation of the miR-9/BRCA1/DNAJB6/KLF4/AKT1 axis in ovarian cancer
Source: Mol Cancer. 2019 Oct 17;18:144. doi: 10.1186/s12943-019-1080-5 (PMC6796346; doi:10.1186/s12943-019-1080-5)
Supplement: Supplementary file 9 — Additional file 9: Figure S6. PLEKHM3 expression is not associated with survivals of ovarian cancer patients. (A) The protein expression of PLEKHM3 was measured by IHC analysis. Nine of eighty-six patients either failed to have a good IHC staining or did not acquired additional FFPE tissue blocks. (B) Kaplan–Meier survival analysis of PLEKHM3 expression in ovarian cancer patients. Differences in the survival risk between the two groups were assessed by the Mantel–Haenszel log-rank test. [file 12943_2019_1080_MOESM9_ESM.pdf]

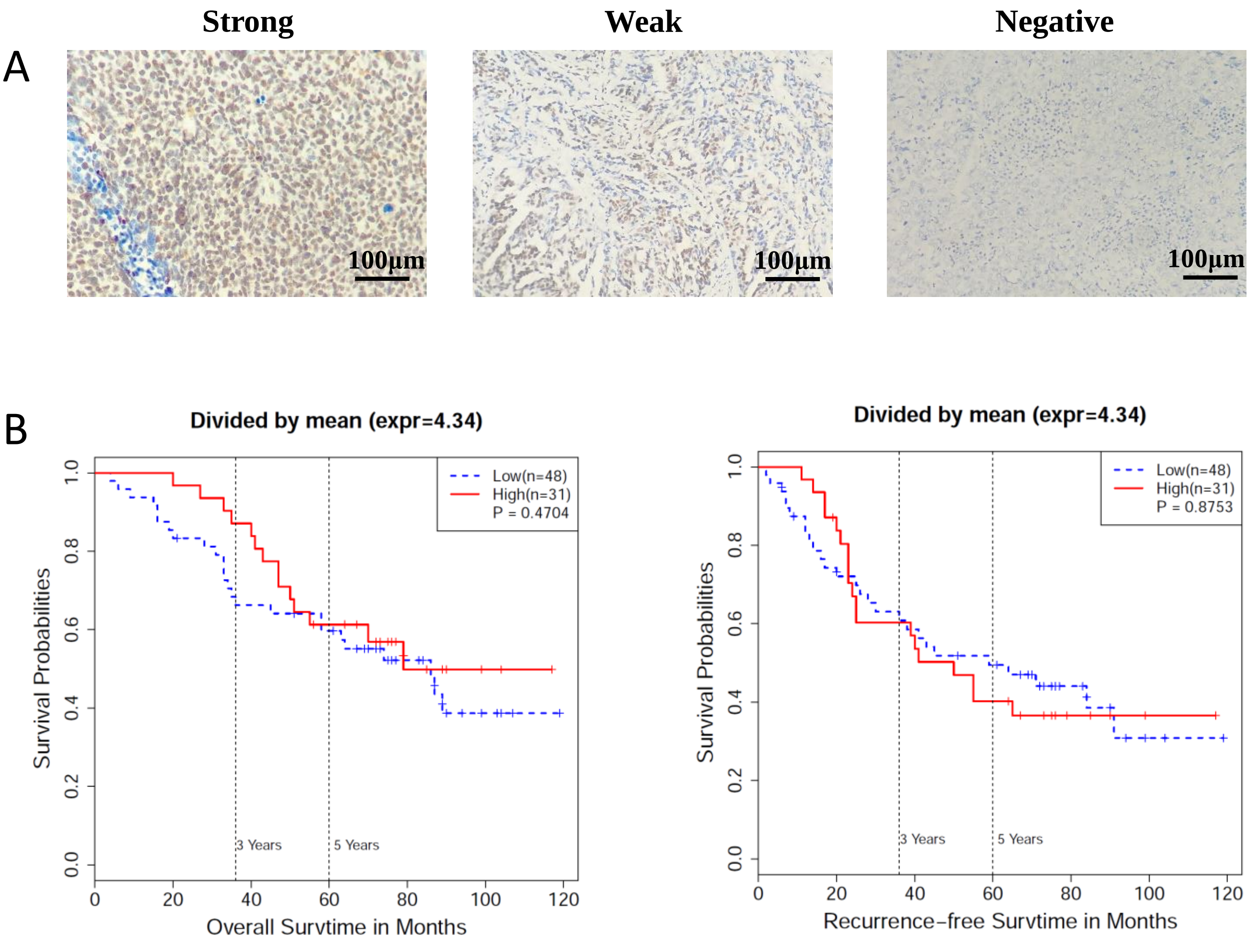

Figure S6. PLEKHM3 expression is not associated with survivals of ovarian cancer patients. (**A**) The protein expression of PLEKHM3 was measured by IHC analysis. 9 of 86 patients either failed to have a good IHC staining or did not acquired additional FFPE tissue blocks. (**B**) Kaplan–Meier survival analysis of PLEKHM3 expression in ovarian cancer patients. Differences in the survival risk between the two groups were assessed by the Mantel–Haenszel log-rank test.
