# Supplementary material for: CircPLEKHM3 acts as a tumor suppressor through regulation of the miR-9/BRCA1/DNAJB6/KLF4/AKT1 axis in ovarian cancer
Source: Mol Cancer. 2019 Oct 17;18:144. doi: 10.1186/s12943-019-1080-5 (PMC6796346; doi:10.1186/s12943-019-1080-5)
Supplement: Supplementary file 10 — Additional file 10: Figure S7. Expression of circPLEKHM3 in ovarian cancer cells. (A) The relative expression of circPLEKHM3 in TOV112D, OVCAR-3, HO8910, MDAH2774, OV90, A2780, and IOSE80 cell lines by real time quantitative RT-PCR. (B) Expression of circPLEKHM3 in single cell clones from A2780 cells. [file 12943_2019_1080_MOESM10_ESM.pdf]

A

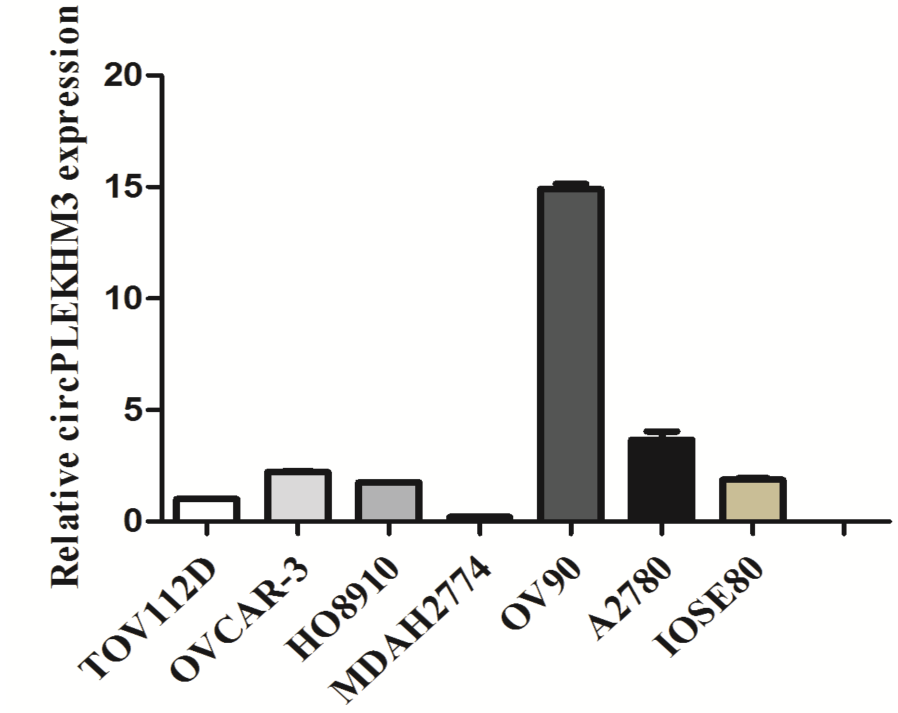

B

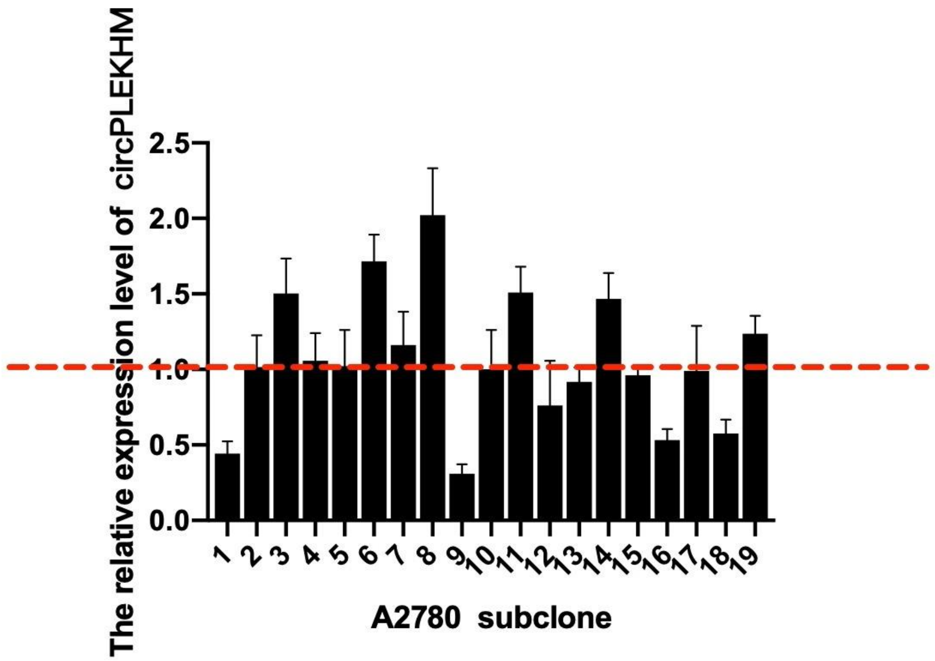

Figure S7. Expression of circPLEKHM3 in ovarian cancer cells. (**A**) The relative expression of circPLEKHM3 in TOV112D, OVCAR-3, HO8910, MDAH2774, OV90, A2780, IOSE80 cell lines by real time quantitative RT-PCR. (**B**) Expression of circPLEKHM3 in single cell clones from A2780 cells.
