# Supplementary material for: CircPLEKHM3 acts as a tumor suppressor through regulation of the miR-9/BRCA1/DNAJB6/KLF4/AKT1 axis in ovarian cancer
Source: Mol Cancer. 2019 Oct 17;18:144. doi: 10.1186/s12943-019-1080-5 (PMC6796346; doi:10.1186/s12943-019-1080-5)
Supplement: Supplementary file 11 — Additional file 11: Figure S8. The relative expression of PLEKHM3 after knockdown or overexpression of circPLEKHM3 in ovarian cancer cells by real time quantitative RT-PCR. [file 12943_2019_1080_MOESM11_ESM.pdf]

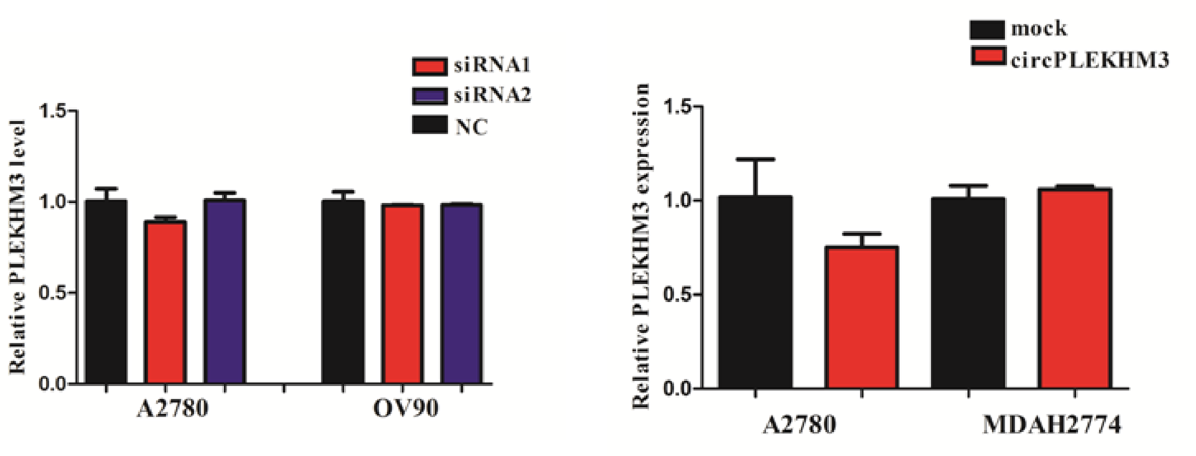

Figure S8. The relative expression of PLEKHM3 after knockdown or overexpression of circPLEKHM3 in ovarian cancer cells by real time quantitative RT-PCR.
