# Supplementary material for: CircPLEKHM3 acts as a tumor suppressor through regulation of the miR-9/BRCA1/DNAJB6/KLF4/AKT1 axis in ovarian cancer
Source: Mol Cancer. 2019 Oct 17;18:144. doi: 10.1186/s12943-019-1080-5 (PMC6796346; doi:10.1186/s12943-019-1080-5)
Supplement: Supplementary file 12 — Additional file 12: Figure S9. Representative images (20× magnification) of immunohistochemical staining for E-cadherin and SNAIL in immunodeficient mice injected with A2780 scramble and shcircPLEKHM3 cells. [file 12943_2019_1080_MOESM12_ESM.pdf]

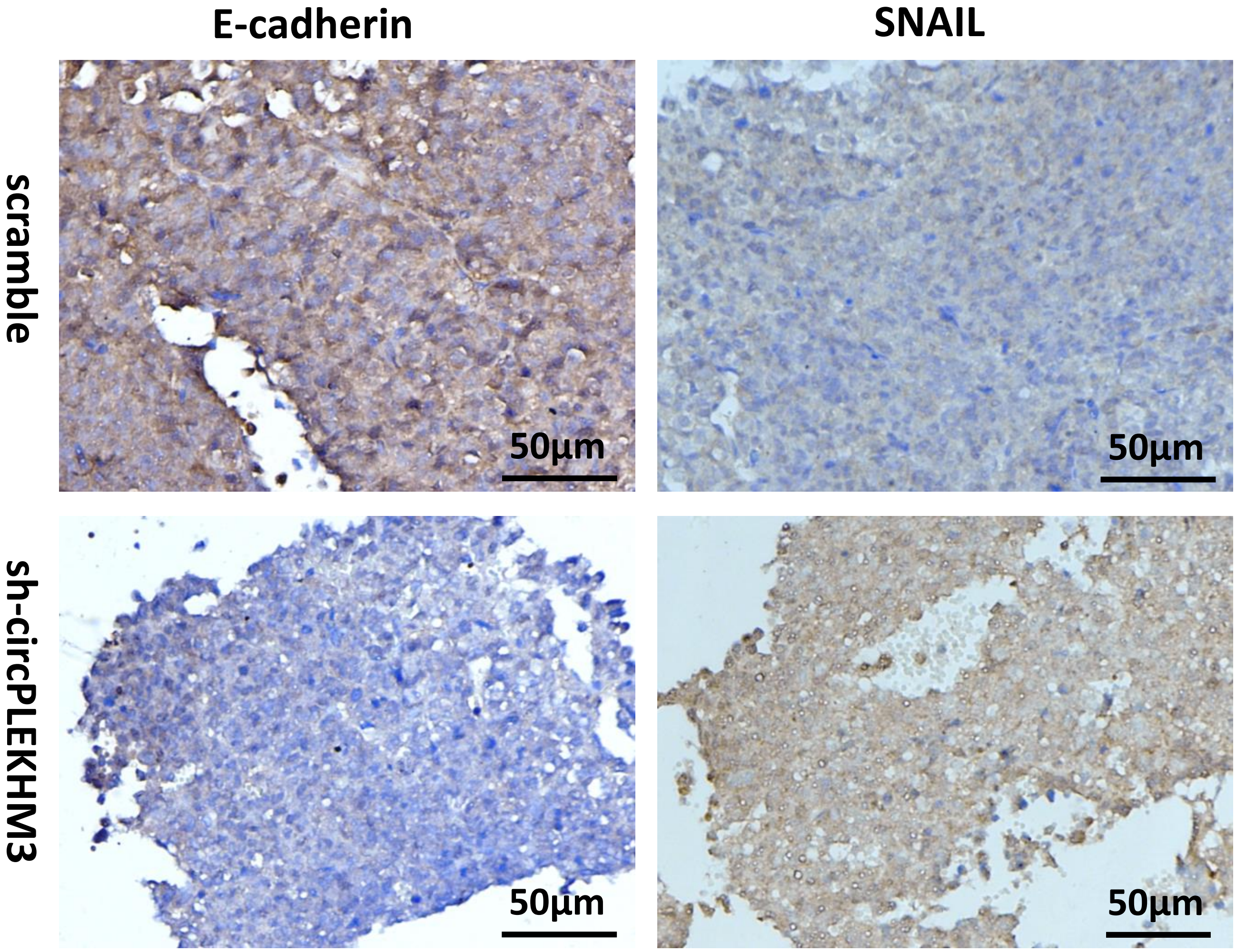

Figure S9. Representative images (20× magnification) of immunohistochemical staining for E-cadherin and SNAIL in immunodeficient mice injected with A2780 scramble and sh-circPLEKHM3 cells.
