# Supplementary material for: CircPLEKHM3 acts as a tumor suppressor through regulation of the miR-9/BRCA1/DNAJB6/KLF4/AKT1 axis in ovarian cancer
Source: Mol Cancer. 2019 Oct 17;18:144. doi: 10.1186/s12943-019-1080-5 (PMC6796346; doi:10.1186/s12943-019-1080-5)
Supplement: Supplementary file 13 — Additional file 13: Figure S10. Sanger sequencing of luciferase report vectors of circPLEKHM3, DNAJB6 variant 1 and KLF4 3′ UTRs. The highlighted sequences represent parts of miR-9 seed sequences that were mutated on psiCHECK™-2 Vectors. [file 12943_2019_1080_MOESM13_ESM.pdf]

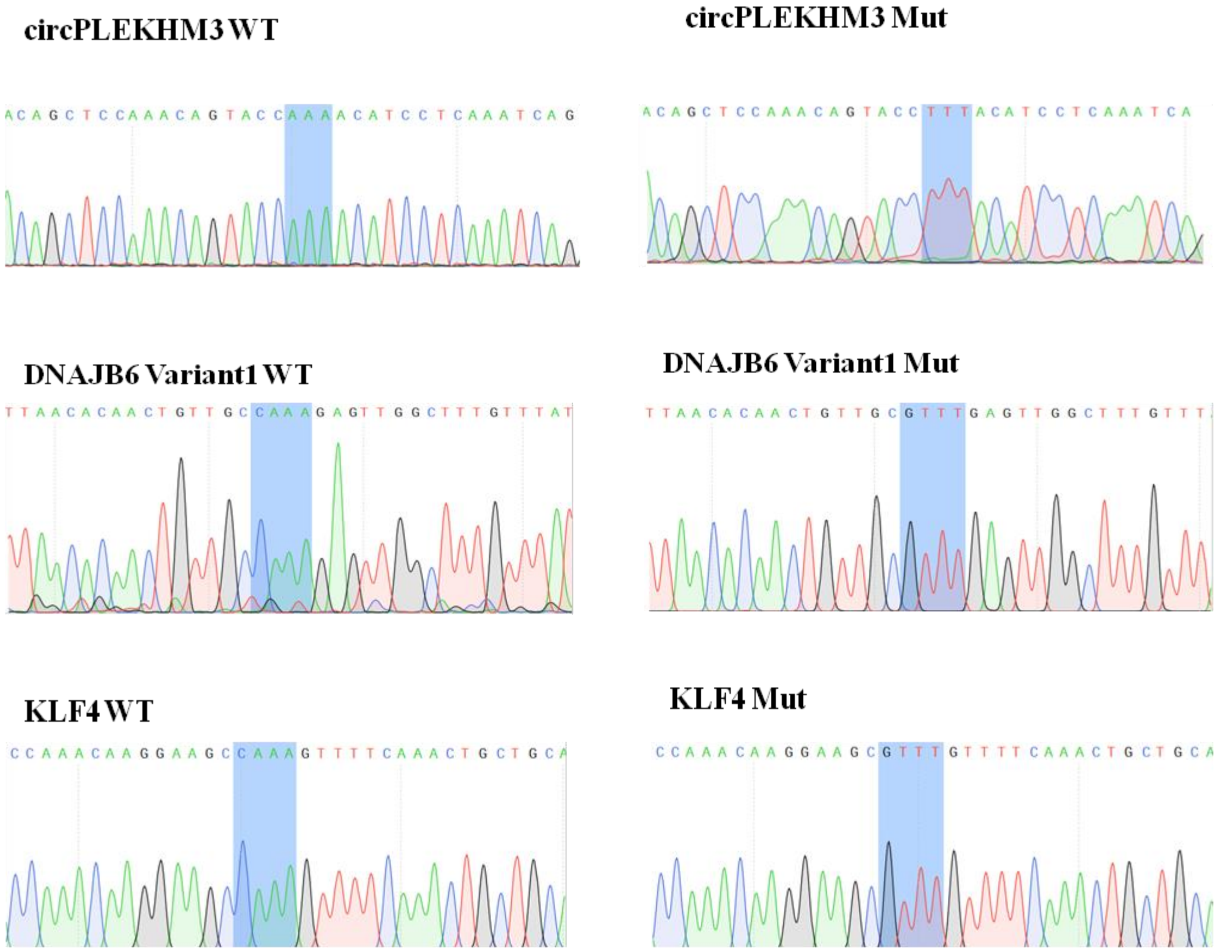

Figure S10. Sanger sequencing of luciferase report vectors of circPLEKHM3, DNAJB6 variant 1 and KLF4 3’ UTRs. The highlighted sequences represent parts of miR-9 seed sequences that were mutated on psiCHECK™-2 Vectors.
