# Supplementary material for: CircPLEKHM3 acts as a tumor suppressor through regulation of the miR-9/BRCA1/DNAJB6/KLF4/AKT1 axis in ovarian cancer
Source: Mol Cancer. 2019 Oct 17;18:144. doi: 10.1186/s12943-019-1080-5 (PMC6796346; doi:10.1186/s12943-019-1080-5)
Supplement: Supplementary file 14 — Additional file 14: Figure S11. The relative expression of KLF4 and DNAJB6 after knockdown of circPLEKHM3 in OV90 cells. The expression of KLF4 and DNAJB6 was quantified by FPKM (fragments per kilobase of exon model per million reads mapped) in the RNA-seq data from OV90 circPLEKHM3 knockdown and negative control (NC) cells. [file 12943_2019_1080_MOESM14_ESM.pdf]

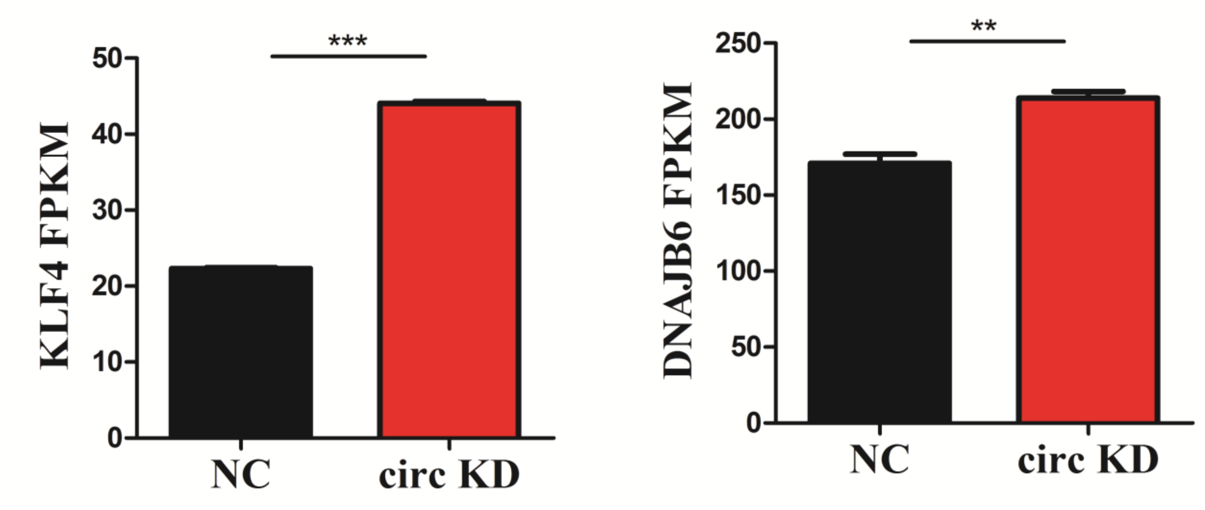

Figure S11. The relative expression of KLF4 and DNAJB6 after knockdown of circPLEKHM3 in OV90 cells. Expression of KLF4 and DNAJB6 was quantified by FPKM (fragments per kilobase of exon model per million reads mapped) in RNA-seq data from OV90 circPLEKHM3 knockdown and negative control (NC) cells.
