# Supplementary material for: CircPLEKHM3 acts as a tumor suppressor through regulation of the miR-9/BRCA1/DNAJB6/KLF4/AKT1 axis in ovarian cancer
Source: Mol Cancer. 2019 Oct 17;18:144. doi: 10.1186/s12943-019-1080-5 (PMC6796346; doi:10.1186/s12943-019-1080-5)
Supplement: Supplementary file 15 — Additional file 15: Figure S12. The relative expressions of DNAJB6a (DNAJB6 isoform a), DNAJB6b (DNAJB6 isoform b), KLF4 and BRCA1 in A2780 cells transfected with miR-9 mimic and inhibitor by immunoblotting analysis. [file 12943_2019_1080_MOESM15_ESM.pdf]

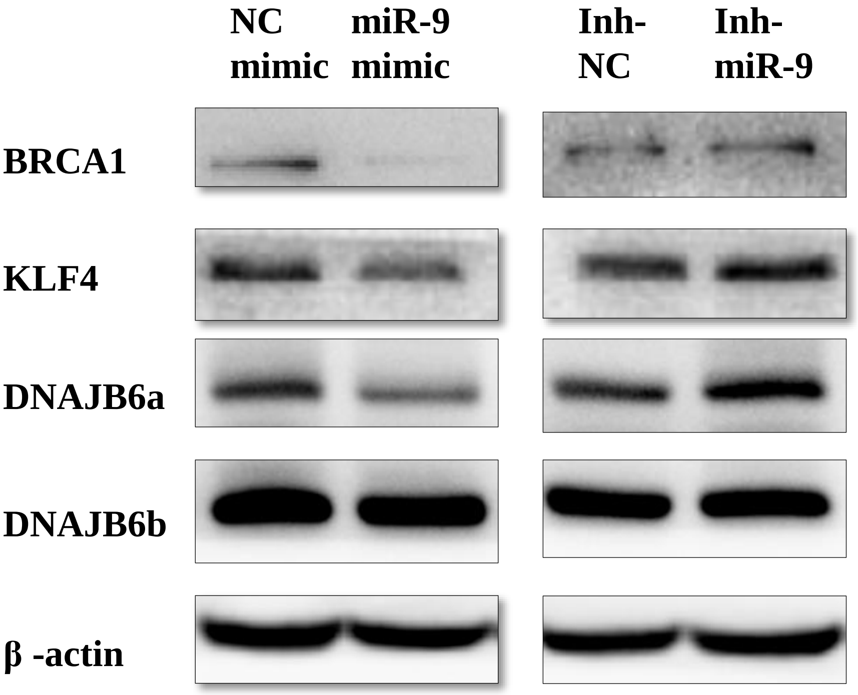

Figure S12. The relative expressions of DNAJB6a (DNAJB6 isoform a), DNAJB6b (DNAJB6 isoform b), KLF4 and BRCA1 in A2780 cells transfected with miR-9 mimic and inhibitor by immunoblotting analysis.
