# Supplementary material for: CircPLEKHM3 acts as a tumor suppressor through regulation of the miR-9/BRCA1/DNAJB6/KLF4/AKT1 axis in ovarian cancer
Source: Mol Cancer. 2019 Oct 17;18:144. doi: 10.1186/s12943-019-1080-5 (PMC6796346; doi:10.1186/s12943-019-1080-5)
Supplement: Supplementary file 16 — Additional file 16: Figure S13. Ovarian cancer patients with a higher expression of DNAJB6 variant 1 and KLF4 are associated with better prognoses. The expression of DNAJB6 variant 1 was retrieved from RNA-seq data of ovarian cancer in TCGA. The expression of KLF4 was downloaded from the Gene Expression Omnibus (GSE3149). Differences in the survival risk between the two groups were assessed by the Mantel–Haenszel log-rank test. [file 12943_2019_1080_MOESM16_ESM.pdf]

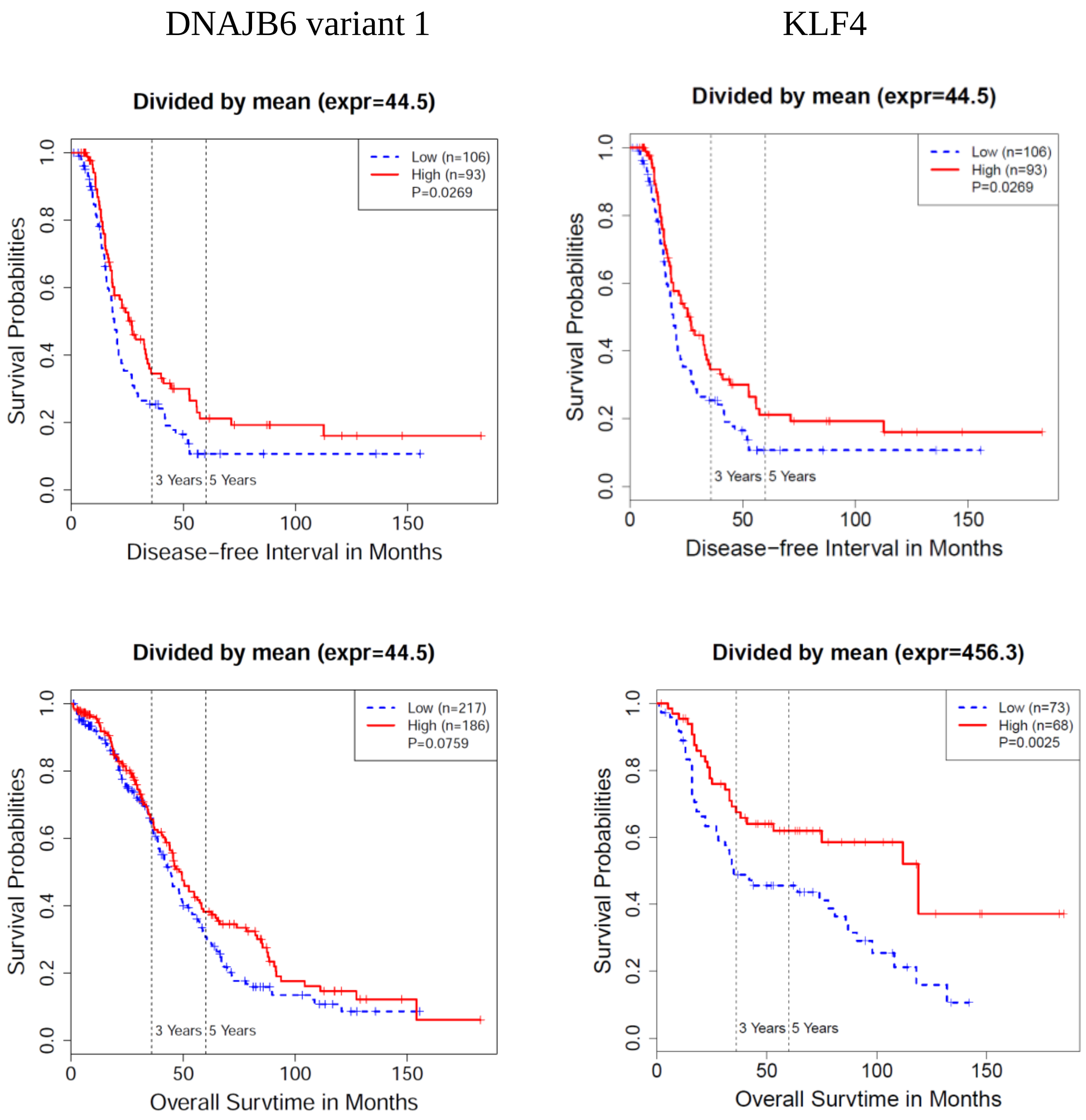

Figure S13. Ovarian cancer patients with higher expression of DNAJB6 variant 1 and KLF4 are associated with better prognosis. The expression of DNAJB6 variant 1 was downloaded from RNA-seq data of ovarian cancer in TCGA. The expression of KLF4 was downloaded from Gene Expression Omnibus (GSE3149). Differences in the survival risk between the two groups were assessed by the Mantel–Haenszel log-rank test.
