# Supplementary material for: CircPLEKHM3 acts as a tumor suppressor through regulation of the miR-9/BRCA1/DNAJB6/KLF4/AKT1 axis in ovarian cancer
Source: Mol Cancer. 2019 Oct 17;18:144. doi: 10.1186/s12943-019-1080-5 (PMC6796346; doi:10.1186/s12943-019-1080-5)
Supplement: Supplementary file 17 — Additional file 17: Figure S14. Apoptosis assays of cells with treatment Taxol and/or MK2206. Cells were treated with Taxol (3 nM) alone, MK2206 (3 μM) alone or Taxol in combination with MK-2206. Cells were harvested and stained using the Annexin V-FITC apoptosis detection kit after about 48 h of treatment. Cells with Annexin V+ staining located in the right upper and lower quadrants were considered as apoptotic cells (mean ± SEM, n = 3). [file 12943_2019_1080_MOESM17_ESM.pdf]

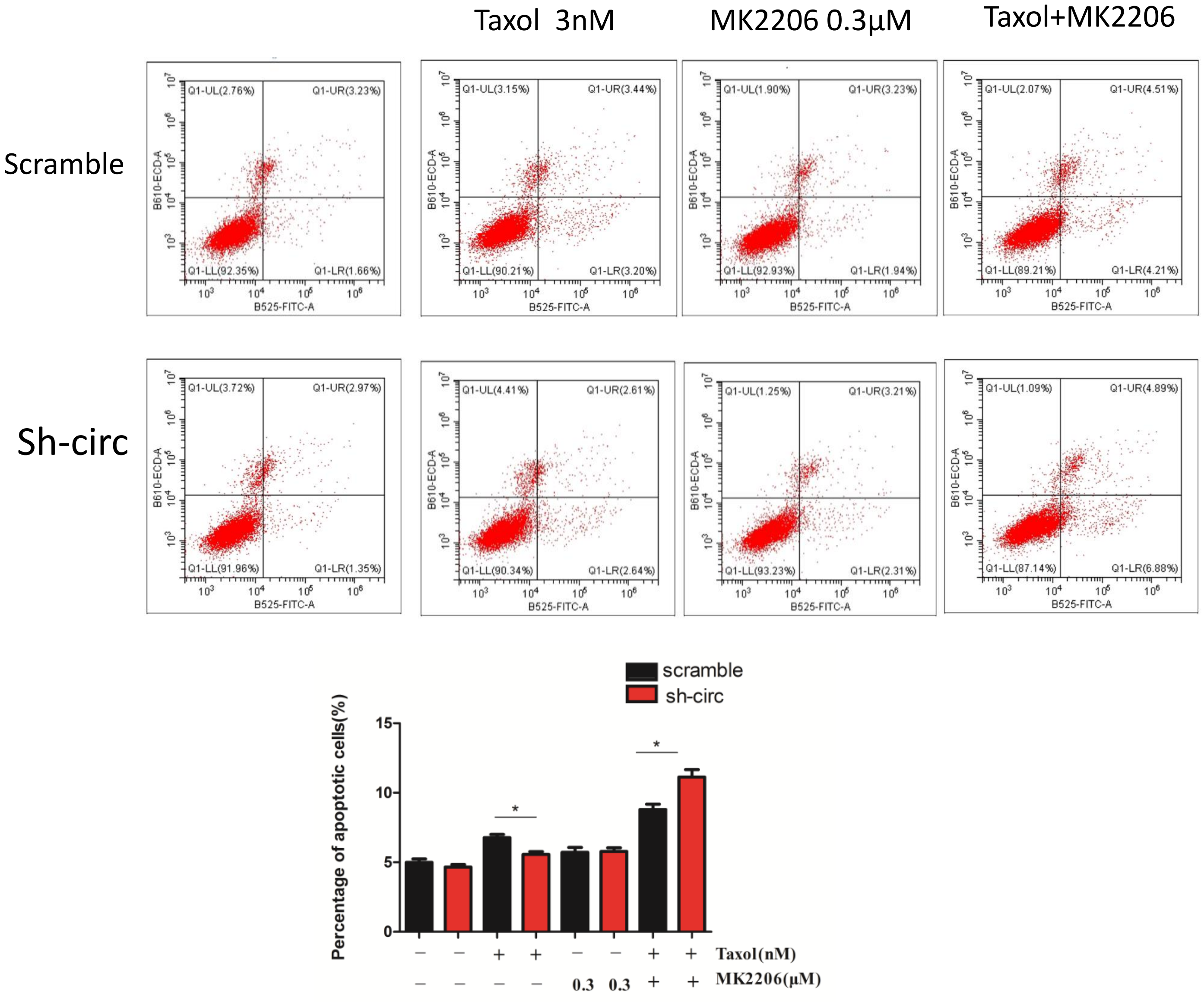

Figure S14. Apoptosis assays of cells with treatment Taxol and/or MK2206. Cells were treated with Taxol (3nM) alone, MK2206 (3μM) alone or Taxol in combination with MK-2206. Cells were harvested and stained using the Annexin V-FITC apoptosis detection kit after about 48 hours of treatments. Cells with Annexin V+ staining located in the right upper and lower quadrants were considered as apoptotic cells (mean± SEM, n = 3).
